# Potential Probiotic *Bacillus subtilis* Isolated from a Novel Niche Exhibits Broad Range Antibacterial Activity and Causes Virulence and Metabolic Dysregulation in Enterotoxic *E. coli*

**DOI:** 10.3390/microorganisms9071483

**Published:** 2021-07-12

**Authors:** Sudhanshu Sudan, Robert Flick, Linda Nong, Julang Li

**Affiliations:** 1 Department of Animal Biosciences, University of Guelph, Guelph, ON N1G 2W1, Canada; ssudan@uoguelph.ca; 2 Biozone, Mass Spectrometry and Metabolomics, Department of Chemical Engineering and Applied Chemistry, University of Toronto, Toronto, ON M5S 3E5, Canada; robert.flick@utoronto.ca; 3 Department of Integrative Biology, University of Guelph, Guelph, ON N1G 2W1, Canada; nongl@uoguelph.ca

**Keywords:** probiotic, bacillus subtilis, antimicrobial, contact inhibition, extreme environment

## Abstract

Microbial life in extreme environments, such as deserts and deep oceans, is thought to have evolved to overcome constraints of nutrient availability, temperature, and suboptimal hygiene environments. Isolation of probiotic bacteria from such niche may provide a competitive edge over traditional probiotics. Here, we tested the survival, safety, and antimicrobial effect of a recently isolated and potential novel strain of *Bacillus subtilis* (CP9) from desert camel in vitro. Antimicrobial assays were performed via radial diffusion, agar spot, and co-culture assays. Cytotoxic analysis was performed using pig intestinal epithelial cells (IPEC-J2). Real time-PCR was performed for studying the effect on ETEC virulence genes and metabolomic analysis was performed using LC-MS. The results showed that CP9 cells were viable in varied bile salts and in low pH environments. CP9 showed no apparent cytotoxicity in IPEC-J2 cells. CP9 displayed significant bactericidal effect against Enterotoxic *E. coli* (ETEC), *Salmonella Typhimurium*, and *Methicillin-resistant Staphylococcus aureus* (MRSA) in a contact inhibitory fashion. CP9 reduced the expression of ETEC virulent genes during a 5 h co-culture. Additionally, a unique emergent metabolic signature in co-culture samples was observed by LC-MS analysis. Our findings indicate that CP9 exhibits a strong antibacterial property and reveals potential mechanisms behind.

## 1. Introduction

Probiotics have gained much interest for the past decade in animal and human health research due to their ability to interact with the host microbiome and modulate cellular functions within the host [1,2]. The International Scientific Association for Probiotics and Prebiotics (ISAPP) defines probiotics as ‘Live microorganisms which, when administered in adequate amounts, confer a health benefit on the host’ [3]. One of the most intriguing host-benefiting properties of probiotics is its antagonism against pathogenic bacteria, which may be attributed to competitive inhibition, promoting growth of commensal or beneficial bacterial, and secretion of antimicrobial secondary metabolites [4,5,6]. In the host, probiotic bacteria may either directly aid exclusion of pathogenic bacteria by production of small antimicrobial compounds [7], or indirectly by strengthening mucosal membranes and modulating the immune capabilities of the host [8]. Various probiotics have been researched in the past; however, their application is limited to the survival of the strains in the intestinal gut microenvironment, which may be influenced by low pH, bile acids, digestive enzymes, host diet, and the colonization-resistant microbiome [9,10,11,12].

Little is known regarding the colonization pattern of probiotics in the context of attachment to the gastro-intestinal tract walls and metabolic interaction with enteric pathogens [10]. In the intestinal microbial landscape, probiotic interaction and communication with the commensal population occurs through metabolic exchange. Previous studies have shown the importance of studying microbial metabolic potential [13,14]. In addition, probiotics are able to cause shifts within the microbiome [15] and thus influence the colonization of enteric pathogens [16,17]. Hence, a more in-depth understanding on the metabolic potential of the probiotics is important in developing and enhancing the efficacy of probiotic interventions.

*Bacillus subtilis* is a gram positive, rod shaped, aerobic or facultative anaerobic bacteria belonging to genus *Bacillus,* widely found in the environment [18]. It has been previously studied and used as a probiotic in fermented foods and also as a supplement [18,19]. One of the unique properties of this bacterium is that it can form spores when challenged with unfavourable conditions for growth [20]. This hardy behaviour may help this bacterial strain to cross the gastrointestinal tract (GIT) fluid barrier and establish itself in the gut. More recently, studies have shown that extreme environment regions, such as deserts and deep oceans, may provide an additional evolutionary benefit to the resident bacteria for survival within the host as well as host adaptation to the environment [21,22]. Therefore, isolation of probiotic bacteria from such niches may allow identification of more robust strains for animal and human supplementation. This notion takes into consideration the fact that microbes living in these extreme conditions are able to overcome the constraints of limited nutrients, desiccation, and extreme fluctuating temperatures. Studying their molecular mechanisms and metabolic interactions with targeted pathogens could further provide cues to predict efficiency for novel antimicrobial probiotic intervention.

We recently isolated a novel *Bacillus subtilis* strain from Sub-Saharan camel [23]. Initial assessment showed a high extracellular protease and cellulase activity of the strain. In the current study, we attempted to test the safety and survival of this novel *Bacillus subtilis* as potential probiotic strain, CP9, in the intestinal environment in vitro, as well as its antagonistic properties against pathogenic bacteria. Moreover, the potential mechanism behind its antimicrobial property was also investigated.

## 2. Materials and Methods

### 2.1. Microbial Strains and Growth Conditions

We previously isolated and characterized *Bacillus subtilis* (CP9; [23]). *Bacillus subtilis* (ATCC 6633) as a control strain was acquired from American Type Culture Collection (ATCC; Edinburg, VA, USA). Enterotoxic *E. coli* (ETEC), *Salmonella typhimurium,* and Methicillin-resistant *Staphylococcus aureus* (MRSA) were acquired from Animal Health lab at University of Guelph, ON, Canada. CP9, ATCC 6633, ETEC, and *Salmonella Typ*. were grown aerobically in LB (Luria-Bertani, St. Louis, MO, USA) medium with constant shaking (200 RPM) at 37 °C. Tryptic soy broth (TSB; Becton–Dickinson, Sparks, MD, USA) was used to grow MRSA with constant shaking (200 RPM) at 37 °C. ETEC strain was positive for virulence factors K88: fimbrial variant 4 (F4), heat-labile enterotoxin A (eltA), heat-labile enterotoxin B (eltB), heat-stable enterotoxin A (estA), and heat-stable enterotoxin B (estB).

### 2.2. Survivability in Gastrointestinal Tract (GIT) Environment

#### 2.2.1. Tolerance to Acid and Bile Salts

The tolerance of CP9 in acidic and bile salts environment was studied by methodology previously described [24] with minor modifications. Briefly, for assessing the tolerance of CP9 to acidic environment, 30 μL of the overnight cultures of CP9 were incubated with 70 μL LB broth adjusted to pH 2, 3, and 6.6 (control) using 1 N hydrochloric acid (HCl) in a 96-well microplate for 2 and 5 h. For assessing the tolerance of CP9 to bile salts environments, 30 μL of the overnight cultures of CP9 were incubated with 70 μL LB broth adjusted with 0% (control), 0.3%, 0.5%, and 1% bile salt (Sigma-aldrich, St. Louis, MO, USA) in a 96-well microplate for 1, 3, and 5 h. After the end of each incubation, cell viability and growth were measured spectrophotometrically via the metabolic activity of the cells using Bacterial Counting Colorimetric Assay Kit (BioVision Technologies, Inc., Chester Springs, PA, USA) following manufacturers protocol. Zero time period in all experiments represented the cellular activity of the initial cell concentration at the time of addition of the substrate. Metabolic cell activity and growth were then compared relative to the zero time point within each treatment group.

#### 2.2.2. Tolerance to Swine GIT Fluids

Swine GIT fluids were collected, as previously described [25] courtesy of Anna Maystrenko. Briefly, the porcine gastrointestinal tract was obtained from the Meat Science Laboratory (University of Guelph, Guelph, ON, Canada). The GIT dissections and collection of gastric, duodenum, and jejunum contents were performed at 4 °C. Digestive contents were centrifuged at 10,000 rpm (9600× *g*) for 10 min at 4 °C, and supernatant fluid was collected, filter-sterilized using Fisherbrand 0.22 μm nylon filter (Fisher Scientific, Waltham, MA, USA) and stored at −80 °C until its use in the Tolerance to GIT fluids experiment. The pH of the collected supernatants was 3.5 and 6.5 for gastric fluid and duodenum fluid, respectively. To assess the tolerance of CP9 in the extracted swine GIT fluids, 30 μL of the overnight cultures of CP9 were incubated with 70 μL of extracted gastric, duodenum, and jejunum fluids in a 96-well microplate for 1, 2, and 5 h. Cell viability and growth were measured using Bacterial Counting Colorimetric Assay Kit (BioVision Technologies, Inc., Chester Springs, PA, USA) following manufacturers protocol. Zero time period in this experiment represented the cellular activity of the initial cell concentration at the time of addition of the substrate. Metabolic cell activity and growth were then compared relative to the zero time point within each treatment group.

### 2.3. Evaluation of Antagonistic Activity of CP9 against ETEC, Salmonella Typ., and MRSA

#### 2.3.1. Agar Radial Diffusion Assay

The inhibitory activity of the CP9 cell-free supernatant (CFS) was evaluated by radial diffusion assay as preciously described [26] with minor modifications. Briefly, 10^8^ CFUs of ETEC, *Salmonella typ.*, and MRSA were mixed with 30 mL of respective nutrient media agar and poured into a 100 mm round Petri dish. With the help of a sterile 1 mL pipette tip, approximately 5 mm diameter holes were punched in the agar and 100 μL of the filter-sterilized cell-free supernatant of CP9 or LB (negative control) or Hygromycin B (10 mg/mL, positive control, Sigma-aldrich, St. Louis, MO, USA) was added to the holes. After the supernatants were fully absorbed, plates were incubated at 37 °C under aerobic conditions. After an incubation period of 24 h, the diameters of the zones of inhibition were observed.

#### 2.3.2. Agar Spot Assay

The contact-dependent inhibitory effect of CP9 was assessed by agar spot assay as previously described [27] with minor modifications. Briefly, 10^8^ CFUs of ETEC, *Salmonella typ*. and MRSA were mixed with 30 mL of respective nutrient media agar and poured into a 100 mm round Petri dish. Overnight cultures of CP9 were grown to log phase until 10^8^ CFUs were achieved and 10 μL of that culture, or LB (negative control) or Hygromycin B (10 mg/mL, positive control) was added to the petri dish with test pathogens. After the spots were fully absorbed, plates were incubated at 37 °C under aerobic conditions. After an incubation period of 24 h, the diameters of the zones of inhibition were observed.

#### 2.3.3. Bacterial Co-Culture Assay

Quantitative analysis of CP9’s inhibitory effect on the test pathogenic strains in a contact-dependent manner was performed by bacterial co-culture assay as previously described [28] with minor modifications. Briefly, 10% of 10^8^ cells of overnight cultures of CP9, ETEC, *Salmonella typ*., and MRSA were inoculated in 5 mL of their fresh respective nutrient media in 15 mL Falcon™ Round-Bottom Polypropylene Test Tubes (Fisher Scientific, Waltham, MA, USA) and vortexed for 10 s. These cultures were named culture A. One ml of the CP9 culture A was mixed with 1 mL of ETEC culture A or *Salmonella typ.* culture A or MRSA culture A in a fresh 15 mL test tube and incubated at 37 °C under aerobic conditions for 5 h. After the end of the incubation, viable cell number of test pathogenic strains were analyzed by performing serial dilutions and colony forming units per ml were counted using pathogen-specific agar plates. MacConkey agar (Thermo Fisher Scientific, Waltham, MA, USA) was used for ETEC and *Salmonella typ.*, Columbia blood agar plates with 5% sheep blood were used for MRSA by counting typical hemolytic colonies, and bacillus cereus agar (PEMBA) with egg yolk and polymyxin B supplement was used for enumeration of CP9.

#### 2.3.4. Cell Line Culture Conditions

The porcine intestinal epithelial cell line, IPEC-J2, originally derived from jejunum of neonatal piglet [29] was acquired from the American Type Culture Collection (ATCC; Virginia, USA). IPEC-J2 cells were cultured in a 1:1 mixture of Dulbecco’s modified Eagle’s medium/Ham’s Nutrient Mixture F-12 (DMEM/F12) supplemented with 10% fetal bovine serum (FBS) and 1% Pen-Strep (10,000 units/mL, Invitrogen, Waltham, MA, USA) under 5% CO2 in a 95% aerobic atmosphere with 90% humidity at 37 °C.

#### 2.3.5. Cell Cytotoxicity Assay

The impact of CP9 on IPEC-J2 cell viability was determined by performing cell cytotoxic assays as described [30]. Briefly, 2 × 10^5^ cells/mL were seeded per well of a 96-well tissue culture plate and grown in 37 °C for 24 h. Media was then replaced with fresh DMEM/F12 media without antibiotics. Cell free supernatant (20, 50, 75, and 100 μL/mL) and CP9 (10^8^ cells/mL) were added to IPEC-J2 cells and incubated at 37 °C for 8 h. Final well volume was 200 μL/well. After the end of incubation, cell viability was analyzed by using alamarBlue™ Cell Viability Reagent (Thermo Fisher Scientific, Waltham, MA, USA) following manufacturer’s instructions.

#### 2.3.6. Cell Surface Adhesion Assay

To determine CP9′s ability to adhere to the IPEC-J2 cells, cell surface adhesion assay was performed as preciously described [30] with minor modifications. Briefly, IPEC-J2 cells were seeded in 12 well tissue plates with 2 × 10^5^ cells/well and grown for 24 h. Cells were then washed two times with PBS to remove the antibiotics in the medium. Fresh DMEM/F12 media without antibiotics was added to all wells. Commercial strain CS and CP9 were grown to log phase and 1 × 10^8^ cells/mL were pelleted, washed with PBS and resuspended in DMEM/F12 incomplete media before incubating with IPEC-J2 cells for 3 h at 37 °C aerobically. After end of incubation, media was removed, and all the wells were washed twice with PBS to remove unadhered bacterial cells. Cells were collected using trypsin-EDTA solution, and serial dilutions were plated on LB nutrient agar plates and incubated aerobically at 37 °C overnight for enumerating and counting adhered bacterial cells.

#### 2.3.7. Gene Expression Analysis

To analyze the effects of CP9 on the expression of virulence-related genes in ETEC, a co-culture experiment was performed where equal volumes of 10^8^ cells of CP9 and 10^8^ cells ETEC or 10^8^ cells of their monocultures were grown in LB nutrient broth aerobically at 37 °C for 5 h. Prior to RNA extraction, RNAprotect Bacteria Reagent (Qiagen 76506) was added to each culture (2:1) for RNA stabilization. Total RNA was then extracted using an RNeasy Protect Bacteria Mini Kit (Qiagen, ON, Canada) according to the manufacturer’s protocol. RNA yield and quality were assessed spectrophotometrically via A230, A260, and A280 nm measurements using a NanoDrop™ 8000 Spectrophotometer (Thermo Fisher Scientific, Waltham, MA, USA). cDNA synthesis was performed as previously described by [31] using a QuantiNova Reverse Transcription Kit (200) (Qiagen, ON, Canada). Quantitative real time-PCR (qPCR) was used to measure the change in the expression levels of transcripts of seven different virulence genes in ETEC, namely motA (motility-flagellar), faeG (adherence-K88, F4, fimbrial protein), tnaA (Tryptophanase-energy metabolism), estA and estB, (heat-stable enterotoxin A and B, respectively), and eltA and eltB (heat-labile enterotoxin A and B respectively), as previously described by [32]. Primers were designed using a Primer-BLAST tool (NCBI; National Center for Biotechnology Information) and synthesized by Integrated DNA Technologies, Guelph, Canada. Primer information is listed in Table S1. The efficiencies of the primers were calculated using CFX Manager Software (Bio-Rad Laboratories Ltd., Hercules, CA, USA). Gene expression was normalized using two reference genes, i.e., the *E. coli* D-glyceraldehyde-3-phosphate dehydrogenase A subunit (gapA) and the *E. coli* 16S ribosomal RNA genes. After determining the threshold cycle (Ct) for each gene, the relative changes in gene expression of ETEC co-cultured with CP9 compared to virulence gene expression of ETEC alone were calculated using the 2^−ΔΔCt^ method in CFX Manager Software (Bio-Rad Laboratories Ltd., Hercules, CA, USA) [32].

### 2.4. Metabolomic Analyses

#### Sample Preparation and LC/MS Procedure

To determine and compare the extracellular metabolite secretions of CP9 in a co-culture with ETEC, a co-culture experiment was performed for five hours. After the end of co-culture incubation, CP9 and ETEC monocultures along with their co-culture samples were centrifuged, supernatant was collected, and filter sterilized using Fisherbrand 0.22 μm nylon filters. LB nutrient media was used as a negative control sample. The samples were immediately frozen in liquid nitrogen and stored in a −80 °C ultrafreezer. Samples were packed in dry ice and shipped to the BioZone Mass Spectrometry Facility in the Chemical Engineering Department at the University of Toronto for metabolite extraction and liquid chromatography-mass spectrometry analysis, courtesy of metabolomics specialist, Robert Flick. Briefly, protein from the samples was precipitated and metabolites were vacuum dried using a speedvac at ambient temperature, followed by resuspension in one tenth the original volume using the appropriate starting solvent for each chromatography method. Samples were then analysed using a Thermo Scientific Ultimate 3000 UHPLC (Thermo Fisher Scientific, Waltham, MA, USA) equipped with a Hypersil Gold C18 column (50 mm × 2.1 mm, 1.9 um) (Thermo Scientific, Waltham, MA, USA) or a Phenomenex Luna NH2 column (150 mm × 2 mm, 3 um), both with guard columns. The temperature of the column was set to 40 °C with a flow rate of 300 μL.min^−1^. Water and acetonitrile containing 0.1% formic acid were used as eluents. The gradient for the C18 column was performed at 5% B for 1 min, linear gradient at 98% B for 6 min, maintained at 98% B for 3 min, returned to 5% B for 0.5 min, and finally a re-equilibration at 5% B for 4.5 min (total runtime 15 min). The gradient for the Luna NH2 column was performed at 90% B for 1 min, linear gradient at 5% B for 4 min, maintained at 5% B for 8 min, returned to 90% B over 1 min, and finally a re-equilibration at 90% B for 6 min (total runtime 20 min). The autosampler of the Thermo Scientific Ultimate 3000 UHPLC was loaded with 10 μL liquid samples. The autosampler temperature was kept at 10 °C. A Q-Exactive Orbitrap mass spectrometer (Thermo Fisher Scientific, Waltham, MA, USA) equipped with a Heated Electrospray Ionization (HESI II) probe was used for compound detection. The system was operated in negative and positive ionization modes for generating spectra. MS1 spectra were acquired over an *m*/*z* range from 80 to 1200 with the mass resolution set to 70 k, AGC Target of 3E6, max injection time 100 ms, spray voltage 3.5 kV, capillary temperature 320 °C, sheath gas 15, aux gas 5, spare gas 2 and s-lens RF level 50. Data-dependent MS2 spectra using a Top5 approach were acquired using a mass resolution of 17.5 k, AGC Target of 1e5, max injection time 50 ms, isolation window of 1.0 *m*/*z* and HCD collision energy of 30. After generating the raw peaks, the untargeted metabolomic data was processed (raw signals exacting, data baselines filtering, peak identification and integration) and metabolite detection (KEGG and BioCyc database) using the differential analysis software package Compound Discoverer 2.1 (Thermo Scientific, Waltham, MA, USA).

### 2.5. Statistical Analyses

All experiments were performed in three biological replicates and data are presented as mean ± standard error of the mean (SEM). For gene expression analysis, experiments were performed in triplicate (*n* = 3) and data are presented as mean ± standard error of the mean (SEM). Data were analyzed with GraphPad Prism v. 7.0 (GraphPad Software, Inc., San Diego, CA, USA) using one-way or two-way ANOVA with Tukey’s post hoc test. *p* < 0.05 was considered significant for all statistical tests.

Metabolomic data was analyzed by performing multivariate statistical analysis and one-way analysis of variance using Metaboanalyst (version 5.0) online analysis software (www.metaboanalyst.ca, accessed 18 October 2020). Briefly, samples were first normalized to the internal control and LB media control. Processed data was filtered to identify and remove any variables followed by normalization and scaling. Principal Component Analysis (PCA) and Partial Least Squares-Discriminant Analysis (PLS-DA) combined with one-way ANOVA and post-hoc analysis were used to screen the significantly differential metabolites. *p* < 0.05 was considered significant for all statistical tests. The model was evaluated by cross validation method using Q^2^ as a performance measure. Clustering and pathway analysis was performed by generating a heat map using Euclidean distances and complete linkage with ANOVA results.

## 3. Results

### 3.1. CP9 Survives Gastrointestinal Environment

**Figure 1:**
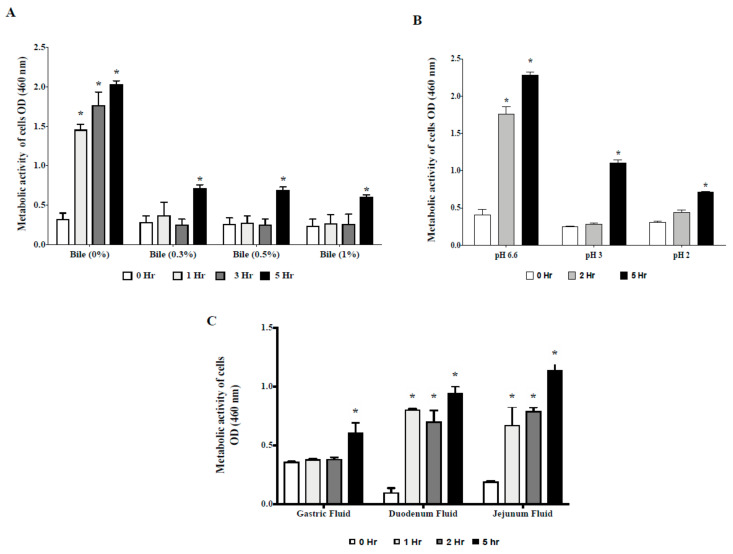
Metabolic activity and viability of CP9 cells in (**A**) bile environment, (**B**) low pH environment, and (**C**) swine gastro intestinal fluids. Data are presented as mean ± standard error of the mean (SEM). Bars with statistical significance denoted as * (*p* ≤ 0.05), using Tukey’s multiple comparison test in ANOVA. Significance in all tests is compared with the initial metabolic activity at time zero within each treatment group. The experiment was performed in triplicates and repeated thrice.

We first examined tolerance of CP9 to different bile salts and pH environments by measuring the metabolically live cell activity as described in *Materials and Method* section. Figure 1A shows that, compared to the initial cell activity at time point zero, no significant change in the cell activity of CP9 was observed in the presence of 0.3%, 0.5% and 1% bile salts for up to 3 h. However, by the end of the 5 h incubation, CP9 metabolic activity increased more than double from the initial 0 h time period across all bile concentrations tested, suggesting significant cell growth of CP9 in the varied bile salt environment after initial adaptation.

Similarly, data from the low pH incubation analysis showed that CP9 maintained its initial metabolic cell activity for up to 2 h of incubation in pH 2 and pH 3 environments (Figure 1B). Metabolic activity of CP9 by the 5 h time period increased significantly (*p* < 0.05) in both pH environments tested, suggesting CP9 could survive in low pH environments after initial adaptation in lower pH.

Overall, results from these suggest that, compared to the untreated CP9 cells, the metabolic activity of CP9 cells in varied concentrations of bile and low pH environments showed a halted growth, and the recovery in the cellular activity in higher time points is indicative of CP9 survival and growth in the stressed environments tested.

To further assess survival of CP9 in the intestinal environment, we incubated CP9 with freshly collected fluid from gastro-intestinal tract (GIT). It was found that, in gastric fluid, CP9 cells maintained a similar metabolic cell activity up to 2 h of incubation, which significantly increased (*p* < 0.05) by the 5 h incubation period (Figure 1C). Similarly, compared to initial metabolic activity at the 0 h time point, CP9 cells incubated with duodenum and jejunum fluids showed enhanced metabolic activity in all time periods tested, suggesting that CP9 may survive and propagate in a GIT environment.

### 3.2. CP9 Adherence and Toxicity on IPEC Cells

**Figure 2:**
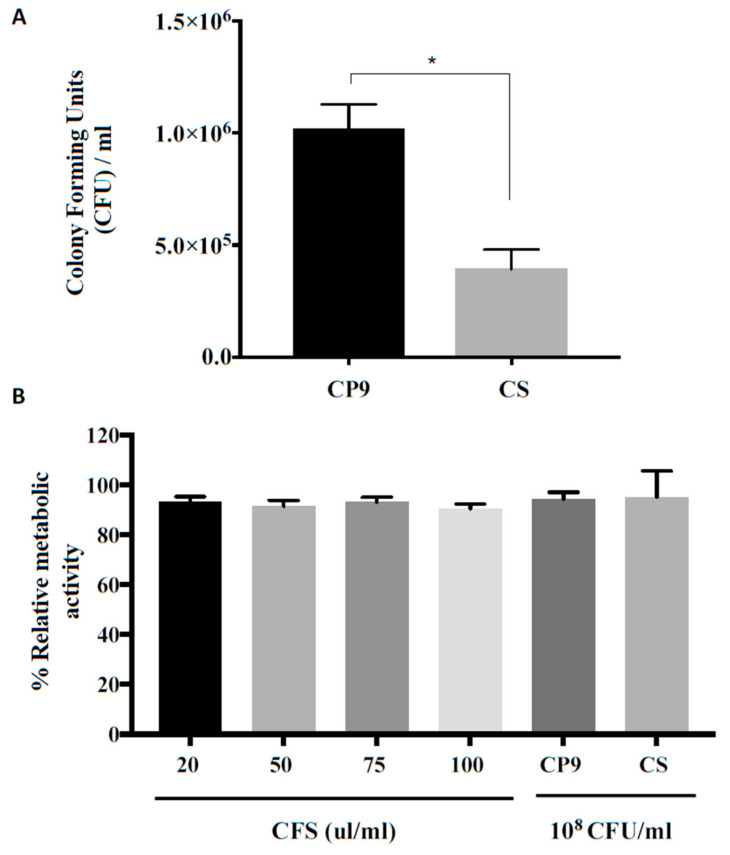
CP9 interaction with Swine intestinal cells, (**A**) CP9 cell surface adherence and (**B**) CP9 impact on swine intestinal epithelial (IPEC-J2) cell viability. Commercially available *Bacillus subtilis* strain (CS) was used as a comparative strain. Data are presented as mean  ±  standard error of the mean (SEM). Bars with statistical significance denoted as * (*p* ≤ 0.05), using Tukey’s multiple comparison test in ANOVA. The experiment was performed in triplicates and repeated thrice.

As a potential probiotic, we next assessed the ability of CP9 to adhere to the pig intestinal epithelial cells, IPEC-J2. To compare the adhesion, we used a commercially available swine probiotic bacillus subtilis (CS) as a control. It was found that CP9 had a significantly higher (*p* < 0.01) adherence to the IPEC-J2 cells, which was 2.6 times higher than the than the commercially available strain CS (Figure 2A).

We next assessed if CP9 impacts intestine cell viability by incubating the CFS of CP9 and the bacterium itself with IPEC-J2 cells for 8 h. As shown in Figure 2B, no significant change in the relative metabolic activity of IPEC-J2 cells was observed when the IPEC-J2 was co-cultured with various concentrations of CP9 CFS or 10^8^ CFU/mL CP9 cells. Similar results were observed for the commercial strain CS, where relative metabolic activity of the cells remained consistently well over 90%. Taken together, these results suggest that CP9 is not cytotoxic to the IPEC-J2 cells and shows better adhesion capacity to the cells compared to commercially available *B. subtilis* strain, CS, in vitro.

### 3.3. CP9 Exhibits Anti-Pathogenic Activity via Contact Inhibition

**Figure 3:**
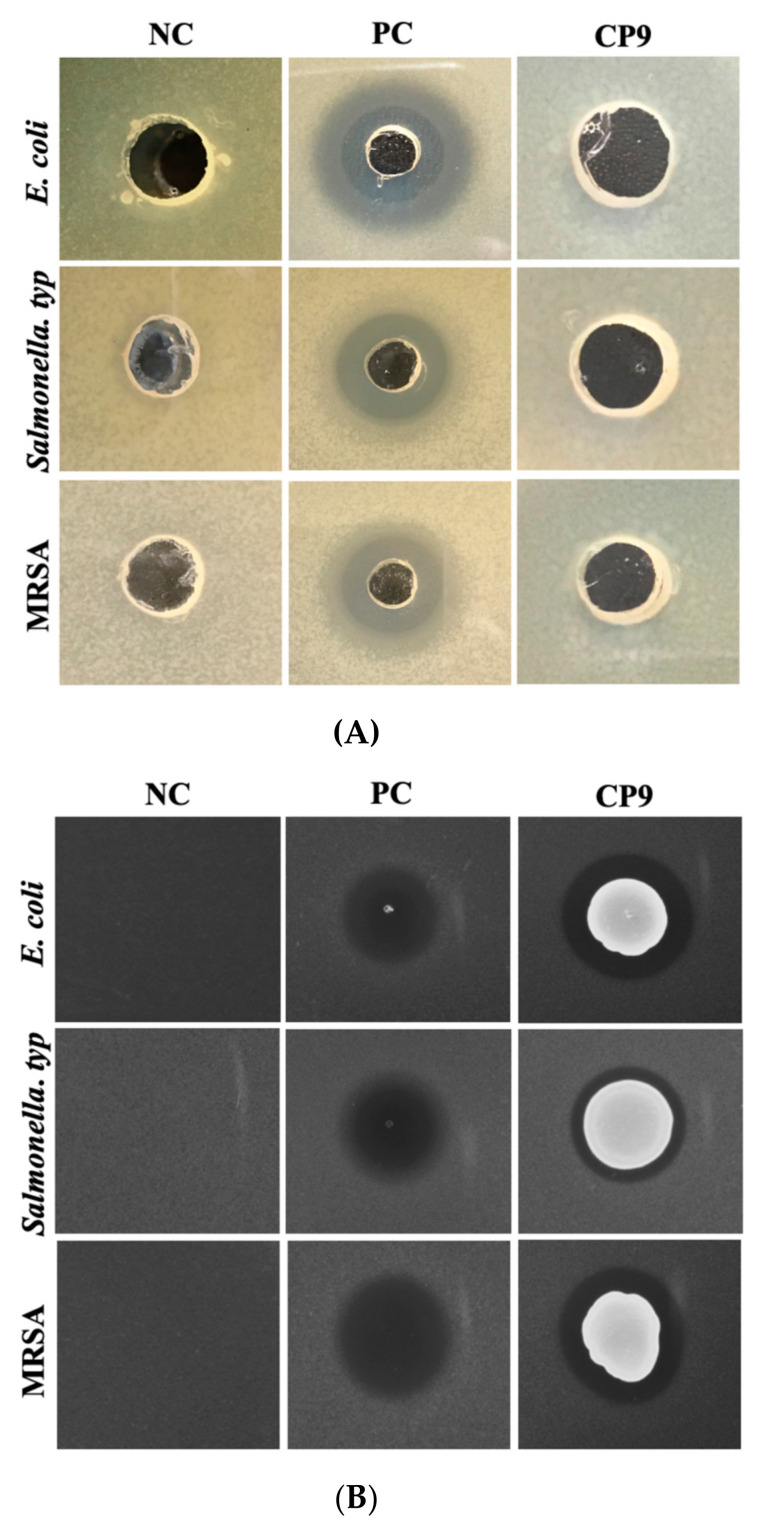

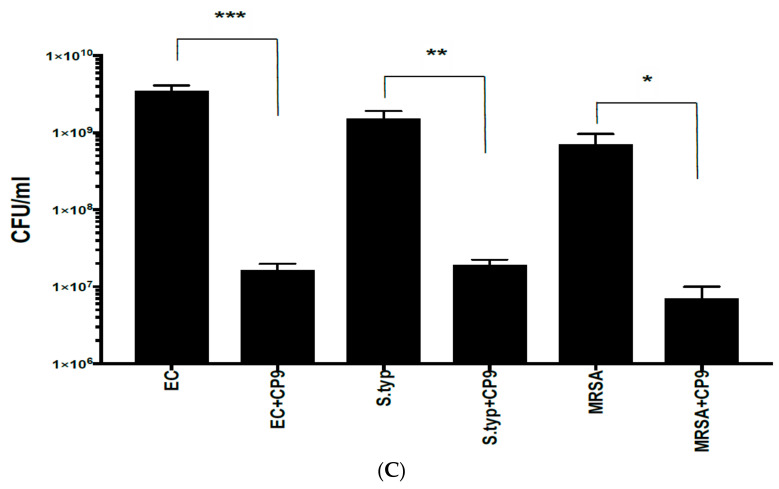
Inhibitory and bactericidal activity of CP9 against ETEC, *Salmonella typ*., and MRSA; (**A**) Agar radial diffusion assay using CP9 cell free supernatant; (**B**) Agar spot assay and (**C**) Co-culture assay. LB media was used as negative control (NC), 10 mg/mL Hygromycin C was used as positive control (PC). Data are presented as mean ± standard error of the mean (SEM). Bars with statistical significance denoted as * (*p* ≤ 0.05), ** (*p* ≤ 0.01) and *** (*p* ≤ 0.001) using Tukey’s multiple comparison test in ANOVA. The experiment was performed in triplicates and repeated thrice.

We first tested the inhibitory potential of CP9′s secretions against ETEC, *Salmonella Typ.* and MRSA. Cell-free supernatant from the log-phase CP9 culture was extracted and inoculated on agar plates containing ETEC, *Salmonella Typ.* and MRSA, separately, using radial diffusion assay. After 24 h of aerobic incubation, no inhibition zone on pathogen growth was observed by the CP9 CFS (Figure 3A), suggesting that CP9 did not secrete anti-pathogen substances in mono-cultures. Interestingly, when CP9 was spotted and grown on the pathogen-inoculated agar plates, clear inhibitory zones were observed in all the pathogens tested (Figure 3B). The results suggest that CP9 may act in a contact-dependent manner against ETEC, *Salmonella Typ.* and MRSA. To further evaluate the impact of CP9 on the viability of pathogenic bacterial strains, we performed a quantitative analysis, where the pathogenic bacterial strains were grown in a co-culture with CP9. Results from the 5 h co-culture experiment showed that, compared to the individual cultures, co-culture with CP9 significantly (*p* < 0.05) reduced the number of ETEC, *Salmonella Typ*. and MRSA (Figure 3C) by more than 75%, further confirming CP9′s ability to halt the growth of pathogenic bacteria when cultured together or in contact with the pathogen.

### 3.4. CP9 Downregulates Virulence Genes Expression in ETEC

**Figure 4:**
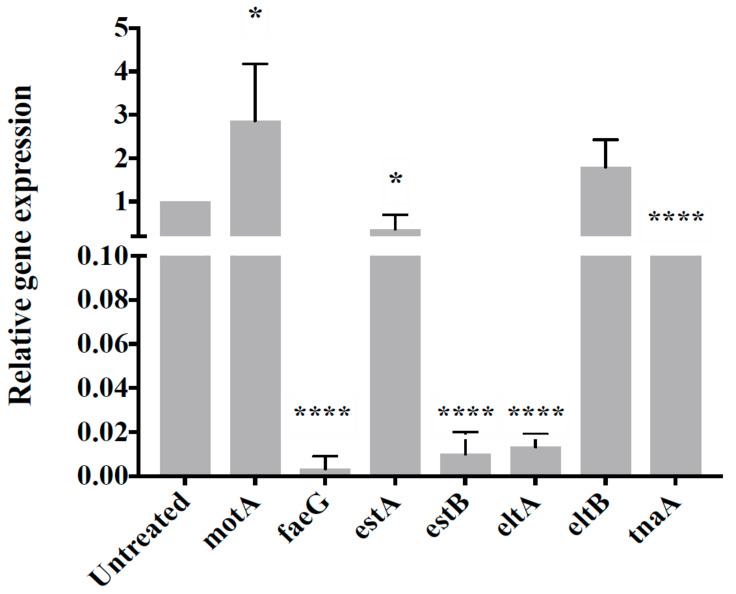
Relative gene expression of ETEC virulence-related genes in co-culture with CP9. Data are presented as mean ± standard error of the mean (SEM). Bars with statistical significance denoted as * (*p* ≤ 0.05), **** (*p* ≤ 0.0001), using Tukey’s HSD test in ANOVA. All values are relative to untreated ETEC monoculture. This experiment was performed in triplicate.

To evaluate if CP9 plays a role in attenuation of ETEC virulence, we analyzed the expression of the several virulence genes of ETEC such as motA (motility-flagellar), faeG (adherence-K88, F4, fimbrial protein), tnaA (Tryptophanase-energy metabolism), estA, estB, (heat-stable enterotoxin A and B, respectively) and eltA and eltB (heat-labile enterotoxin A and B respectively). As shown in Figure 4, while the expression of adherence gene faeG was downregulated upon co-culture with CP9, there was an increased expression of motA gene responsible for flagella motility. Expression of genes encoding ETEC toxins, estA, estB, and eltA, were significantly downregulated upon co-culture with CP9; however, there was no significant change observed in the expression of eltB gene. Finally, expression of tnaA gene was also seen significantly downregulated upon incubation with CP9. Overall, these data suggest that CP9 influenced and suppressed the expression of ETEC toxin genes and genes involved in pathogen adherence.

### 3.5. Secreted Metabolic Repertoires of the CP9 and ETEC Co-Culture Vary Significantly Than Their Monocultures

**Figure 5:**
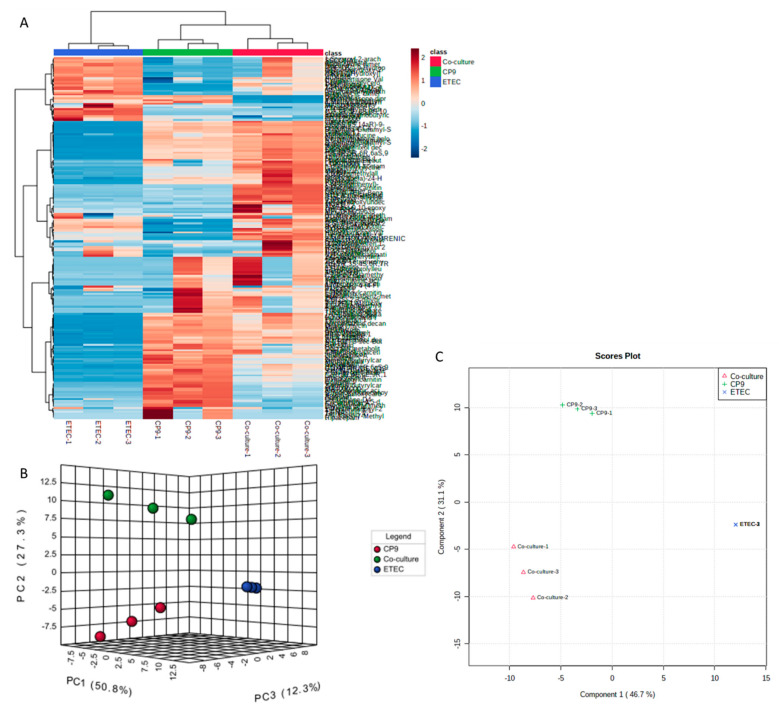
Metabolic repertoire of emergent features in CP9 and ETEC co-culture. (**A**) Heat map of successful annotated compounds showing significant clustering patterns between mono and co-culture of CP9 and ETEC; (**B**) 3D score plot of PCA model of variance and (**C**) Score plot of PLS-DA model of variance showing clear separation between mono and co-culture metabolomic profiles of CP9 and ETEC. Detail list of the metabolites are provided in Supplementary Table S2. Statistical analysis was performed using MetaboAnalyst software v4.036, ANOVA testing with Fisher’s post hoc analysis plus false discovery rate (FDR) analysis. Features with *p* < 0.05 plus fold change of >2 were considered significant.

In order to decipher the metabolic impact of bacteria-bacteria interaction and potential mechanisms on growth inhibition in a co-culture, we performed metabolomic analysis on secreted factors in the CFS of mono and co cultures of CP9 and ETEC, using liquid chromatography coupled to a mass spectrometer (LC-MS). An untargeted metabolomics approach was applied to capture a wide array of secreted metabolites in mono versus co-culture groups. The metabolomic features were first normalized and refined by using the same culture media as previously described [16]. The system successfully identified 199 metabolites (Table S2), which were then statistically analyzed through Metaboanalyst (version 5.0) online analysis software. Compared to the mono-cultures, the co-culture had substantially altered metabolomic profiling, as seen via heat map and clustering analysis (Figure 5A). To compare the metabolomic patterns in the secretions of CP9, ETEC and their co-culture, we first performed PCA and PLS-DA multivariate statistical analyses to evaluate the metabolic features that caused significant separation between the groups. Figure 5 shows clear separation in metabolomic profiles between the three groups. The R2 and Q2 values obtained from PLS-DA were >0.8 (Figure S1), suggesting that the models used were of reasonable and acceptable quality and could be further used for analyzing significant differences between the groups. Interestingly, samples from co-culture appeared to be located closer to the CP9 mono-culture, suggesting that the co-culture metabolome was less resembling to the negatively affected strain ETEC (Figure 5B,C). Importantly, the emergent separation of metabolomic profiles in the co-culture suggests that the interaction of CP9 and ETEC may have resulted in production of specific metabolites that may play a role in negatively affecting ETEC growth in the co-culture.

### 3.6. CP9 and ETEC Co-Culture Induces Emergence of New Metabolites with Antimicrobial Properties

To determine the significantly different metabolic features between the groups, we combined the Variable Importance for the Projection (VIP, VIP obtained by PLS-DA model) data with *p*-value obtained by performing one-way ANOVA analysis on the identified metabolites. For determining significant differential metabolites, the metabolite had to pass the screening criteria of VIP > 1 and *p* < 0.05 as previously described by [33]. A total of 143 metabolites were found to be significantly distinct between the mono- and CP9 and ETEC co-cultures (Table S3), and 82 differential metabolites were found to have a VIP score above 1 (Table S4). In order to look for the unique metabolites that may be secreted or influenced by CP9 in response to co-culture with ETEC, we focused on (i) the metabolites that emerged only as a result of co-culture and were absent in the monocultures, (ii) the metabolites that emerged in CP9 mono-cultures and were overexpressed in co-culture and (iii) the metabolites that had significantly higher concentrations in ETEC monoculture but were either suppressed or overexpressed in the co-culture (Table 1). Out of the 31 selected metabolites (Table 1), 11 metabolites presented only in the co-culture group. The unique profile consisted of metabolites belonging to fatty acid and energy metabolism, tryptophan metabolism, polyamine metabolism, nitrogen metabolism, and secondary metabolites with known antimicrobial properties. In the second group, 14 metabolites already observed in the CP9 monoculture group were seen to be increased significantly (*p* < 0.05) in the co-culture group. These unique metabolites belonged to fatty acid and energy metabolism, glutathione metabolism, polyamine metabolism and cell-cell signaling, nucleoside analogues, arachidonic acid metabolite, serine protease inhibitor and secondary bacterial bile acid. Additionally, three metabolites with structural similarities to commercial drugs/chemicals emerged in second group; however, no exact match in the metabolomic database or relevance to their role in microbial physiology could be determined. Lastly, the third group, reflecting metabolites that were present in ETEC and were significantly influenced by CP9 in co-culture, showed varied abundance of metabolites involved in tryptophan metabolism, secondary metabolites involved in ETEC virulence, purine metabolism, and cell growth.

## 4. Discussion

Over the past couple decades, probiotics have been researched for their unique antagonistic properties to pathogenic bacteria. They may achieve this by various mechanisms such as competitive exclusion and secretion of small antibacterial molecules. Our study evaluated properties of a novel *Bacillus subtilis* strain, CP9, for its probiotic and antimicrobial potential in vitro and identified unique small molecules during CP9-ETEC interaction.

Gastrointestinal tract in vitro-mimicking models have been widely and successfully used for testing the passage survival and colonization of the probiotic strains [34]. In our study, CP9 showed significant resistance in the GIT environment conditions in vitro, which is a positive trait of a potential probiotic bacteria, since the colonization and persistence of probiotic bacteria in the GIT is an important factor for exerting a beneficial effect on the host [35]. We observed an initial adaptation of CP9 in the low pH and varied bile environments (Figure 1A,B), which was consistent to a previous study, where *Bacillus subtilis* cultures were seen to adapt initially to varied pH and alkaline stress before recovering growth rapidly [36].

*Bacillus subtilis’* existence is ubiquitous in the environment and has been shown to be found in symbiotic existence within plants and animal kingdom [37]. Due to its spore-forming practical edge over the other vegetative forms of probiotics, it has gained a substantial research interest in human and animal consumption and is generally considered safe due its long history of consumption [18]. However, due to the strain-specific properties, behavior, and interactions in the mammalian intestinal tract, the toxigenic potential of a novel probiotic strain is an inevitable check point [38]. In the current study, CP9 showed no cytotoxicity to the swine intestinal epithelial cells and was consistent with the previous studies performed on *Bacillus subtilis*-based probiotics [39,40]. Furthermore, CP9 showed a higher adherence to the IPEC-J2 intestinal cells than a commercially available probiotic *Bacillus subtilis*. Intestinal adherence is an important determining factor for probiotics to modulate a host’s immune system as well as competitively prevent the adhesion of opportunistic and pathogenic enteric bacteria [41,42].

It has been shown that CFS of *Bacillus subtilis* can inhibit enteric pathogens such as ETEC and *Salmonella typ.* [43,44]. However, in our study, CP9 appeared to display an antimicrobial effect via contact-dependent inhibition and, in parts, via metabolic influence. The absence of anti-pathogenic activity in the CP9 cell-free supernatant suggests the absence of toxins or antimicrobial molecules in the monocultures of CP9. Interestingly, upon contact with the pathogenic strains ETEC, *Salmonella*, and MRSA, a substantial decrease in the pathogenic cell growth was observed, which may suggest, in parts, activation of pathways for CP9′s cellular response to pathogens. Highly competitive bacteria survive by using their exploiting (nutrient depletion) and/or interfering (release of antagonistic factors) abilities to survive in heavily populated environments [45]. As part of the interference mechanism, contact-dependent inhibition (CDI) describes the bacterial adjustment of internal cellular responses and cell differentiation pathways in response to external cue [45,46]. Upon sensing interbacterial competition, members of the same microbial community can ramp up their cellular growth, activate the secretion system and deliver the regulatory factor across membrane upon contact with the competitor strain. These regulatory factors can influence the cellular processes of the competitor strains and inhibit their cell growth [47,48]. Both gram-positive and gram-negative bacteria have been shown to utilize their secretion system for CDI to influence cellular growth of competitor strains. For example, *Bacillus subtilis* have been shown to utilize the CDI secretion system to secrete and deliver toxic polymorphic protein regulatory factors to influence morphological changes and growth inhibition in target strains such as *E. coli* [49,50]. Contact-dependent growth inhibition is also profoundly used by gram-negative bacteria, such as *E. coli*, for delivering toxins to the neighbouring target cells [46,51], however, since we did not observe contact inhibition from the ETEC on CP9 in our study may suggest that CDI growth inhibition of ETEC was driven by CP9.

Enteric pathogens such as ETEC express various virulence factors that are regulated by the environment and help ETEC outcompete its rival commensals in the GIT and evade host defenses such as motility (*motA*, flagellar movement), adherence (*faeG*, F4, fimbrial protein), heat-stable enterotoxins (*estA*, *estB*), heat-labile enterotoxins (*eltA*, *eltB*), and tryptophanase (*tnaA*, virulence regulator and energy metabolism) [52,53,54]. Interestingly, when co-cultured with ETEC, CP9 significantly downregulated the expression of the virulence genes that are responsible for adherence, faeG and toxin-releasing genes *estA*, *estB*, *eltA.* The finding that flagellar motility gene motA, one of the ETEC virulence genes, was overexpressed during co-culture was surprising. The significance of the increase expression is currently unknown. Interestingly, it has been reported that overexpression of motA is associated with reduced ETEC cell growth [55].

Energy metabolism is vital for physiological processes and biochemical pathways for driving division and cell growth in microbes such as bacteria [56,57]. Interactions in mixed microbial cultures are driven by metabolite exchanges and are dependent on symbiotic and sometimes competitive behaviours [58,59]. Tryptophan and its metabolic derivatives such as indole, indole derivatives, and kynurenic acid are vital for bacterial protein synthesis and cell growth [60]. In ETEC, tryptophan metabolism is executed by enzyme tryptophanase (*tnaA*) and its expression is tightly regulated by external tryptophan availability [61,62]. Importantly, in ETEC, pathogenicity and virulence have also been shown to be regulated by the *tnaA* gene [32,52,63]. In our study, exposure to CP9 downregulated the ETEC *tnaA* gene in the co-culture, which was also reflected in the lower kynurenic acid levels observed in the ETEC group (Table 1). In addition to these results, higher abundance of its downstream metabolite, 8-Methoxy kynurenic acid, in the co-culture samples might suggest depletion of tryptophan from the media by CP9 as part of competitive exclusion, a typical strategy for survival in mixed microbial cultures for its own growth. This notion is further supported by reduced growth of ETEC in co-culture and abundance of downstream metabolites of tryptophan such as, indole and its derivatives seen in the co-culture, suggesting an external tryptophan utilization by CP9 for its rapid growth and production of survival proteins (Table 1). Interestingly, microbially derived indole and its derivatives, known for their antimicrobial effects [64], have been previously shown to negatively regulate virulence of GIT pathogens, such as enterohemorrhagic *Escherichia coli* (EHEC) and *Citrobacter rodentium* [65,66]. Consistent to our study, Singh et al. 2014 previously found that in the co-culture with *Bacillus subtilis,* there was a higher indole yield, the number of *E. coli* decreased dramatically compared to its monoculture, and *Bacillus subtilis* in co-culture [67]. This might suggest, in parts, the antibacterial effect of the *Bacillus subtilis* derived indole and its derivatives seen in our study. However, the study by Singh and colleagues was an experimental demonstration of indole production in the co-culture using mathematical modelling and function of time. Therefore, results from our study should be taken carefully as further experiments may be needed to model and quantify the depletion of tryptophan and production of its downstream metabolites such kynurenic acid and indole by individual strains in the co-culture, especially when *E. coli* is also shown to produce indole under stress conditions [68]. This could additively represent the indole production in the co-culture in our experiment.

In mixed microbial cultures, competitive exclusion is achieved by either rapid nutrient utilization for energy and protein production for cellular growth, by secreting antimicrobial metabolites, or by both [69]. Endogenous and exogenous fatty acid metabolisms play a critical role in energy derivation, protein synthesis, transport for cellular growth, and survival in bacterial physiology [70,71]. Our finding on the significant emergence of carnitine, acyl carnitines, and other fatty acid metabolites in the co-culture samples reflects a rapid metabolism of fatty acid for intracellular transport and energy production [72,73,74] in the co-culture samples. Additionally, there was significant emergence of polyamine putrescine and its intermediate, N(1)-acetylspermidine, which are responsible for regulating virulence factors for survival and cellular growth in stressful environment in eukaryotes including *Bacillus subtilis* and *E. coli* [75,76] in co-culture samples. This may further suggest stimulation of the stress response between CP9 and ETEC [75,76] in the co-culture samples. The decrease in ETEC cells in co-culture and emergence of the co-culture metabolic features appearing closer to the CP9 samples in PCA and PLS-DA plot are suggestive of the notion that production of these metabolites may be driven by CP9 for its defense, rapid cellular growth to outcompete and weaken ETEC. Furthermore, metabolites that appeared in high concentrations in ETEC mono-cultures were significantly regulated in the co-culture, suggesting that CP9 may influence ETEC cellular metabolism and growth. For example, secondary metabolite gamma-Aminobutyric acid (GABA), which is responsible for spore germination, bile and low pH resistance, and tight regulation of virulent factors in enterotoxic and enteropathogenic *E. coli* [77,78], was observed in significantly lower abundance in the co-culture compared to ETEC mono-culture. This data is further supported by the lower cell number of ETEC after the co-culture. Similarly, di-peptide gln-gln involved in ETEC acid resistance [79] and arabinosylhypoxanthine involved in the purine metabolism, *E. coli* cellular growth, and virulence in mixed culture [80,81] were seen in higher abundance in the co-culture group. This may reflect an initial defensive response of ETEC to CP9 in the co-culture. However, as explained above, it should be noted that our study did not analyze the emergent metabolomic profile as a measure of the production or consumption of metabolites by either of the strains. Our study is in agreement with previous research by Medlock and colleagues, where, through metabolic modelling, it was shown that in mixed culture pairings, co-culture metabolomic profiles were less similar to the negatively impacted strain than the other strain, and the emergent metabolic profile of co-culture was directly correlated to the abundance of the highly competitive strain in the culture [16]. This notion is further supported by the emergence of unique antimicrobial secondary metabolites in co-culture and CP9 samples (Table 1) respectively, that may have synergistically impacted the growth of ETEC in co-culture. For example, valclavam, which is a metabolite of clavam class of β-lactam antibiotics, has been shown to strongly inhibit pathogenic *E. coli* blocking methionine biosynthesis [82]. However, to our knowledge, these have only been shown to be produced by *Streptomyces antibioticus* spp. [83,84]. Hence, emergence of valclavam in the co-culture warrants further investigation to analyze if its biosynthesis was triggered by CP9. Similarly, we observed a unique presence of leukotriene C4 and leukotriene E3 in CP9 metabolome samples and their significant abundance in the co-culture (Table 1). Leukotrienes are inflammatory mediators and are formed by oxidation of arachidonic acid by lipoxygenase enzyme. They are traditionally known to be exclusively produced in mammalian leukocytes for defense against microbial infections [85]. Interestingly, lipoxygenase activity, which was historically thought to be of eukaryotic function, has recently been found in various bacterial species [86]. This opens the door for further investigation into the presence of lipoxygenase activity in CP9 that may have resulted in the biosynthesis of leukotrienes in our study. This will be particularly important, as none of the probiotic classes of bacteria have been shown to possess this enzyme activity, which could have a direct impact on host immune response towards pathogenic microbes. Another unique metabolite significantly expressed in the co-culture samples was 3-Hydroxymyristic Acid, which is the most common fatty acid constituent of the lipid A component of bacterial lipopolysaccharides (LPS) [87]. Its significant presence in the co-culture suggests that CP9 may have caused the lysis of ETEC. This notion is supported by a previous study where *Bacillus subtilis* was shown to sensitize and lyse *E. coli* cells, which was driven by its proteolytic activity [88]. Alternatively, ETEC may have released LPS as an initial stress response in co-culture with CP9; however, this has a low probability since we did not observe any growth inhibitory effect of the ETEC CFS or co-culture CFS on CP9 growth (Data not shown). Lastly, emergence of an increased expression of melagatran, a serine protease inhibitor in the co-culture, is intriguing, since in gram negative bacteria such as *E. coli,* serine protease is secreted via autotransporter pathway and are implicated in expression of virulence and direct pathogenicity of its infection [89]. This could be a possible way CP9 may have downregulated the expression of ETEC virulence factors observed in our study. Overall, these results suggest how these unique metabolites may regulate the interactions between CP9 and ETEC by influencing their metabolic pathways and secreting secondary metabolites in the mixed culture either to weaken the opponent or depleting the essential nutrients for cell growth. However, these results warrant further investigation into the biosynthesis and purification of these metabolites to determine the extent of the impact they might have on antagonistic potential of CP9.

In conclusion, our study showed a strong antibacterial effect of potential probiotic, *Bacillus subtilis* CP9, which was driven by a contact-dependent mechanism of inhibition. We also found a substantial survival rate of CP9 in GIT fluids. However, since GIT fluids can vary in composition and pH depending on the diet of the animal, more tests need to be performed by utilizing GIT fluids from pigs or animals in question fed varied diets. Our study further revealed that CP9 successfully downregulates the virulence factors in the ETEC on a molecular level upon direct contact, which may be one of the possible mechanisms of CP9′s antagonistic potential. However, whether this effect is translated on a protein level was not analyzed in this study and warrants further investigation. Interspecies interactions within the gut are highly complex and impacted by metabolic cooperation and competitiveness [59,90]. Therefore, a detailed understanding about mechanisms of interactions of novel probiotic strains with gut pathogens may likely improve the predictability of the biological effect of the probiotic. Our study used an untargeted, data-driven approach to identify metabolic patterns that may influence bacterial growth in ETEC and CP9 co-culture, and proposed mechanisms that may contribute to the appearance of these patterns. However, this study did not analyze the biosynthesis and substrate utilization by either of the strain in co-culture. Perhaps incorporating a metabolic model that analyzes biosynthesis and utilization of these metabolites over time could provide normalized behaviors of the CP9 and ETEC metabolic patterns in co-culture. Developing such a model and validating these experiments will require a much larger data set than used in the current study. Nonetheless, extension of our approach to time-coursed metabolic modelling will provide more specific insights into CP9-induced growth inhibition of ETEC as well as other pathogenic bacteria. There is an increasing interest in developing novel probiotic-based interventions for animal and human use. However, traditional methods have primarily been focused on characteristics based on survival and properties of the probiotic strains. We envision that our study may provide the basis of preliminary understanding into the complex interaction of probiotic bacteria with an enteric pathogen, laying the foundation for the potential application of the probiotic for animal and human use.

## Figures and Tables

**Table 1:** Differential metabolites uniquely emerging in co-culture and mono-cultures.

VIP Scores					
Group	Metabolite	Comp. 1	Comp. 2	*p* Value	Pathway/Function
Co-culture only	9- Decenoylcarnitine	1.3611	1.3578	5.69 × 10^−8^	Fatty acid/energy Metabolism
	Carnosine	1.412	1.3994	1.47 × 10^−6^	Fatty acid/energy Metabolism
	5-Methoxy-3- indoleaceate	1.3661	1.3571	2.08 × 10^−6^	Tryptophan metabolism and antimicrobial metabolite
	Indole	1.5536	1.5057	1.70 × 10^−6^	Tryptophan metabolism and antimicrobial metabolite
	Valclavam	1.3586	1.3543	4.26 × 10^−6^	Antimicrobial metabolite
	3-[(3- Hydroxyundecanoyl)oxy]-4- (trimethylammonio) butanoate	1.3429	1.3343	0.00092045	Fatty acid/energy Metabolism
	n-phenethyl acetamide	1.4326	1.4083	0.0011712	Antibacterial secondary metabolite
	LT9970000/ Furmecyclox	1.4475	1.4108	0.0029438	Drug
	Uric Acid	1.039	1.0652	0.0034036	Nitrogen metabolism/Amino acid and protein synthesis
	Putrescine	1.0175	1.0174	0.0071156	Polyamine/Cell growth and metabolism/ Virulence
	MFCD00059633/ 3- Hydroxymyristic Acid	1.0336	1.0203	0.023742	Bacterial metabolite/fatty acid metabolism
CP9 and overexpressed in Co-culture	C8-Carnitine	1.4194	1.4042	1.22 × 10^−9^	Fatty acid/energy Metabolism
	L-Cysteinylglycine disulfide	1.5642	1.5136	2.15 × 10^−9^	Di-peptides/ Glutathione metabolism
	N(1)- acetylspermidine	1.4443	1.4248	1.82 × 10^−8^	Polyamine metabolite/Cell- Cell signalling/ Virulence
	5,6- Dihydrothymidine	1.4407	1.3837	4.66 × 10^−8^	Nucleoside analogues
	Leukotriene C4	1.4209	1.3659	7.24 × 10^−8^	Arachidonic Acid metabolite/ antimicrobial
	Naloxegol	1.4683	1.4096	3.51 × 10^−7^	Drug
	gamma-Glu-gln	1.4715	1.4125	3.67 × 10^−7^	Glutathione metabolism
	Aderbasib	1.5448	1.4852	1.11 × 10^−6^	Drug
	Spironolactone	1.5097	1.4494	2.27 × 10^−6^	Drug
	3-[(2,6- Dimethylheptanoyl)oxy]-4- (trimethylammonio) butanoate	1.4724	1.4402	5.38 × 10^−6^	Fatty acid/energy Metabolism
	Leukotriene E3	1.262	1.247	6.88 × 10^−6^	Arachidonic Acid metabolite/ antimicrobial
	Carnosine.1	1.4944	1.4362	0.00012389	Fatty acid/energy Metabolism
	Melagatran	1.5172	1.4729	0.00015017	Serine protease inhibitor
	(3beta,5beta)-24- Hydroxy-24- oxocholan-3-yl beta-D-glucopyranosiduronic acid	1.4674	1.4101	0.0010426	Secondary bacterial bile acid metabolite/ antibacterial metabolite
ETEC and over/ under expressed in Co-culture	Kynurenic acid (↓)	1.2825	1.2397	7.61 × 10^−8^	Tryptophan metabolism
	gamma- Aminobutyric acid (↓)	1.2656	1.2242	9.81 × 10^−8^	Spore germination/bile and low pH resistance
	8- Methoxykynurenic acid (↑)	1.3368	1.3216	1.05 × 10^−5^	Tryptophan metabolism
	Gln-Gln (↑)	1.2812	1.2803	0.00010342	L-Glutamine Di- peptide/acid resistance
	(1Z,3R,5E,8S,9S, 10R)-N-[(Z)-2-(3- Chloro-4- hydroxyphenyl) vinyl]-3,9- dihydroxy-2,4- dimethoxy-6,8,10- trimethyl-7-oxo-5- tetradecenimidic acid (↑)	1.3569	1.3068	4.29 × 10^−7^	Unknown
	Arabinosylhypoxanthine (↑)	1.1994	1.1915	1.07 × 10^−7^	Purine metabolism/E. coli cellular growth and virulence in mixed culture

## Data Availability

The data presented in this study are available on request from the corresponding author.

## Supplementary Materials

The following are available online at https://www.mdpi.com/article/10.3390/microorganisms9071483/s1, Figure S1: Cross Validation analysis for predicting PLS-DA model accuracy, Table S1: Genes and primer sequences used for ETEC reference genes and virulence-related genes, Table S2: Metabolomic profiles of co-culture and mono-cultures of CP9 and ETEC, Table S3: Significant metabolites emerging in Co-culture and mono-cultures of CP9 and ETEC, Table S4: Variable Importance in projection (VIP) scores across PLS-DA components indicating scale of variable metabolite concentration in mono-cultures and co-culture of CP9 and ETEC.
