# Supplementary material for: Potential Probiotic Bacillus subtilis Isolated from a Novel Niche Exhibits Broad Range Antibacterial Activity and Causes Virulence and Metabolic Dysregulation in Enterotoxic E. coli
Source: Microorganisms. 2021 Jul 12;9(7):1483. doi: 10.3390/microorganisms9071483 (PMC8307078; doi:10.3390/microorganisms9071483)
Supplement: Supplementary file 1 [file microorganisms-09-01483-s001.zip › Figure S1.pdf]

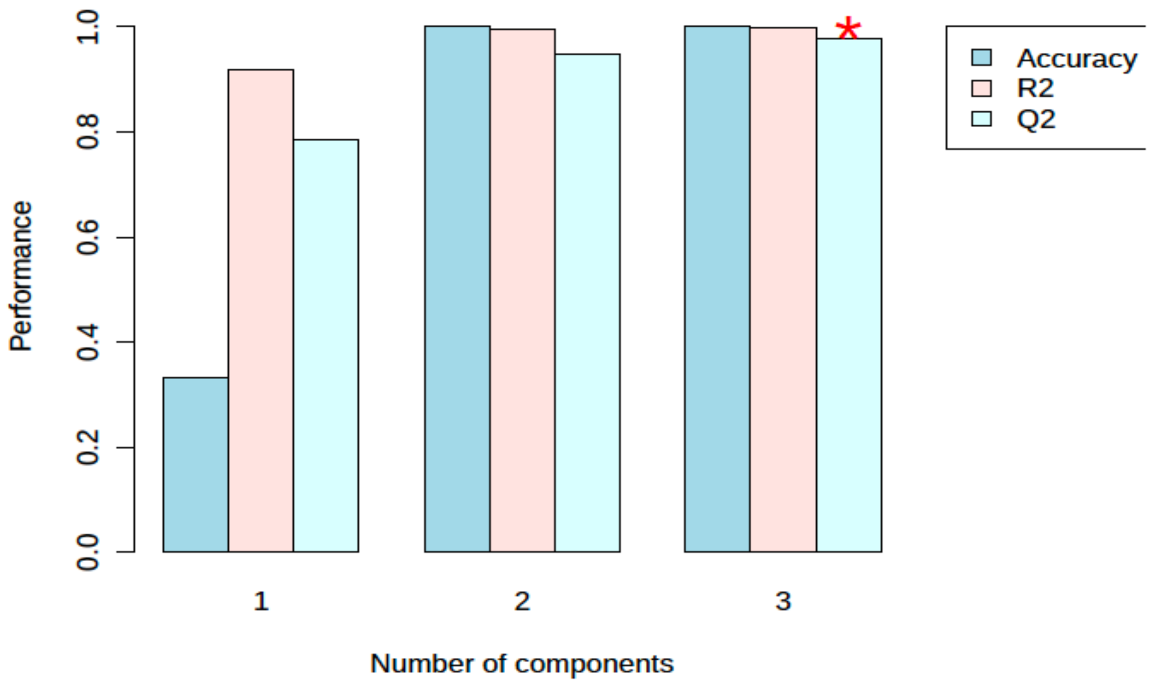

Figure S1. Cross Validation analysis for predicting PLS-DA model accuracy using different number of components in Metaboanalyst. The red star indicates the best classifier.
