# Supplementary material for: Potential Probiotic Bacillus subtilis Isolated from a Novel Niche Exhibits Broad Range Antibacterial Activity and Causes Virulence and Metabolic Dysregulation in Enterotoxic E. coli
Source: Microorganisms. 2021 Jul 12;9(7):1483. doi: 10.3390/microorganisms9071483 (PMC8307078; doi:10.3390/microorganisms9071483)
Supplement: Supplementary file 1 [file microorganisms-09-01483-s001.zip › Table S1.pdf]

Table S1. Genes and primer sequences used for ETEC reference genes and virulence-related genes.

Gene	Primer Sequence
faeG	F: ACGTCGCAGGTTCTTACAGG<br>R: GCTCCACTGAGTGCTGGTAG
eltA	F:TTGGTGATCCGGTGGGAAAC<br>R:AGGAGGTTTCTGCGTTAGGTG
eltB	F:GAGCGGCGAAACATTTCAGG<br>R:GGTATTGCCTCCTCTACCGC
estA	F:CAACTGAATCACTTGACTCTT<br>R:TTAATAACATCCAGCACAGG
estB	F:TGGTTGCAGCAAAAGGATGC<br>R:GTCCGTCTTGCGTTAGGACA
motA	F:TCGTTCACGCGTTAGGTTCA<br>R:TCATTTTGCTGGTTTCGGCG
tnaA	F:CGCCAAGAAAGATGCGATGG<br>R:CGTCATACAGACCTACCGCC
gapA	F:GATGGCCCGTCTCACAAAGA<br>R:CAGACGAACGGTCAGGTCAA
16S	F:GGCGCATACAAAGAGAAGCG<br>R:CTCCAATCCGGACTACGACG
