# Supplementary material for: Potential Probiotic Bacillus subtilis Isolated from a Novel Niche Exhibits Broad Range Antibacterial Activity and Causes Virulence and Metabolic Dysregulation in Enterotoxic E. coli
Source: Microorganisms. 2021 Jul 12;9(7):1483. doi: 10.3390/microorganisms9071483 (PMC8307078; doi:10.3390/microorganisms9071483)
Supplement: Supplementary file 1 [file microorganisms-09-01483-s001.zip › Table S2.pdf]

Table S2. Metabolomic profiles of co-culture and mono-cultures of CP9 and ETEC as successfully identified by LC-MS platform and their contribution to the first principal component (PC1) and the second principal component (PC2).

METABOLITES	PC1	PC2
3-hydroxydodecanoylcarnitine	-0.078849	-0.079838
Surfactin C	-0.068909	0.00036935
Mavik	-0.096919	0.011056
Naloxegol	-0.091815	0.047312
11343172	-0.034824	0.081351
Rizatriptan	-0.074296	0.063392
Hydrocortisone Valerate	0.0070218	0.11409
(7S,13aS)-7-Methyl-5,8,13,13a-tetrahydro-6H-isoquinolino[3,2-a]isoquinolinium	-0.041088	-0.082741
3-[(3-Hydroxyundecanoyl)oxy]-4-(trimethylammonio)butanoate	-0.034395	0.12152
(1S,2R,3S,4S,6R,7R,8R,14S)-3-Hydroxy-2,4,7,14-tetramethyl-9-oxo-4-vinyltricyclo[5.4.3.0~1,8~]tetradec-6-yl {[2-methyl-1-(valylamino)-2-propanyl]sulfanyl}acetate	-0.064893	0.027028
LT9970000	-0.058901	0.087222
2-methylbutyrylcarnitine	-0.081049	-0.071223
SECONAL	0.073857	0.069134
Enviradene	-0.09434	0.012825
Lithocholic acid taurine conjugate	-0.093585	0.0044306
2949	-0.057367	0.047739
N-[(10Z)-3-sec-Butyl-7-isobutyl-5,8-dioxo-2-oxa-6,9-diazabicyclo[10.2.2]hexadeca-1(14),10,12,15-tetraen-4-yl]-1-methylprolinamide	-0.095047	0.0036649
Ripazepam	-0.041005	-0.08298
nylon cyclic dimer	0.086655	0.044673
4-Methylcarbostyril	-0.00089223	-0.13543
(2'S)-Deoxymyxol 2'-alpha-L-fucoside	0.056513	0.10098
7-Sulfocholic acid	-0.089497	-0.048126
Prednisolone tebutate	-0.069435	0.043731

**Tributyl phosphate**	-0.066667	-0.0086081
**1872050**	-0.063171	0.023536
**1-Stearoyl-2-arachidonoyl-sn-glycero-3-phosphate**	0.064888	0.078759
**Cifenline**	0.08272	0.053192
**N-(tert-Butoxycarbonyl)-L-leucine**	-0.073518	-0.087858
**2,2,6,6-Tetramethyl-1-piperidinol (TEMPO)**	-0.064745	0.024566
**Arg-Trp**	-0.077191	-0.073915
**Leukotriene C4**	-0.095375	0.036632
**Traumatic Acid**	-0.063886	-0.029368
**Astemizole**	-0.096563	0.0041761
**L-Hexanoylcarnitine**	-0.091096	-0.044876
**Atosiban**	-0.071872	-0.085928
**(-)-Lupinine**	-0.063233	0.030043
**(Hydroxyethyl)methacrylate**	-0.056523	-0.10873
**chivosazole A**	-0.089902	-0.044004
**1-(4-Butylphenyl)-6,6-dimethyl-1,6-dihydro-1,3,5-triazine-2,4-diamine**	-0.076132	0.071463
**2-Amino-9,10-epoxy-8-oxodecanoic acid**	-0.039301	0.09217
**4-Hydroxyprolylleucine**	-0.063738	0.027487
**2-methylbutyrylcarnitine.1**	-0.081803	-0.06838
**Leukotriene E3**	-0.042512	0.084478
**Atagabalin**	-0.065029	0.017867
**D-gamma-Glutamyl-S-[(5Z,8beta,12E,15S)-1,15-dihydroxy-1,11-dioxoprosta-5,12-dien-9-yl]-L-cysteinylglycine**	-0.090149	0.051589
**Bisacodyl**	-0.081731	0.053085
**imiquimod**	-0.08008	-0.017375
**Levulinic acid**	-0.0554	-0.11052
**Leu-Gly-Pro**	-0.082153	-0.048758
**(3S,6S,9S,14aR)-9-[(2S)-2-Butanyl]-6-[(1-methoxy-1H-indol-3-yl)methyl]-3-(6-oxooctyl)decahydropyrrolo[1,2-a][1,4,7,10]tetraazacyclododecine-1,4,7,10-tetrone**	-0.092191	0.05094
**Dexamethasone beloxil**	-0.092004	0.039398
**Zuclopenthixol decanoate**	-0.090936	0.051114
**Guanine**	-0.081654	-0.07415
**[(2R,3R,4E,6E,9R,11S,12S,13S,14E)-2-Ethyl-9,11,13-trimethyl-8,16-dioxo-12-{[3,4,6-trideoxy-3-(dimethylamino)-beta-D-xylo-**	-0.092417	-0.037648

**hexopyranosyl]oxy}oxacyclohexadeca-4,6,14-trien-3-yl]methyl 6-deoxy-beta-D-allo pyranoside**		
**perphenazine decanoate**	-0.09393	0.012737
**Caprolactam**	-0.046527	0.066669
**trilobolide**	-0.091079	-0.017826
**Melagatran**	-0.061914	0.10067
**(1S,4R,5R,6R,6aS,9S,9aE,10aR)-1,5-Dihydroxy-9-(hydroxymethyl)-3-isopropyl-6,10a-dimethyl-1,2,4,5,6,6a,7,8,9,10a-decahydrodicyclopenta[a,d][8]annulen-4-yl alpha-D-glucopyranoside**	-0.09528	-0.016996
**3-[(2,6-Dimethylheptanoyl)oxy]-4-(trimethylammonio)butanoate**	-0.051078	0.11445
**(Hydroxyethyl)methacrylate.1**	0.085115	0.05047
**Methionylleucine**	-0.0753	0.064585
**Pulcherriminic acid**	-0.065064	0.061191
**Tetraneurin A**	-0.042656	-0.085255
**Adipic acid**	-0.057612	0.034273
**1-O-[(3alpha,5beta,7alpha)-3,7-Dihydroxy-24-oxocholan-24-yl]-beta-D-galactopyranose**	-0.088062	0.039938
**Embelin**	-0.06031	0.018187
**GLIMEPIRIDE, CIS-**	-0.092726	-0.028311
**Callichiline**	-0.08491	0.061214
**methyprylon**	-0.06428	0.030099
**(2R)-1-[(Hydroxy{[(1s,3R)-2,3,4,5,6-pentahydroxycyclohexyl]oxy}phosphoryl)oxy]-3-(palmitoyloxy)-2-propanyl (5Z,8Z,11Z)-5,8,11-icosatrienoate**	0.083549	0.05429
**(Hydroxyethyl)methacrylate.2**	-0.025916	0.075598
**spironolactone**	-0.087582	0.059459
**(Z)-Norendoxifen**	-0.08592	0.025457
**Darifenacin**	0.024362	-0.12912
**(3beta,5beta)-24-Hydroxy-24-oxocholan-3-yl beta-D-glucopyranosiduronic acid**	-0.078689	0.069361
**Ala-Tyr**	-0.097168	-0.00016088
**Betamethasone dipropionate**	0.042927	-0.11994
**Choline**	0.072452	0.064716
**(1S,4R,5R,6R,6aS,9S,9aE,10aR)-1,5-Dihydroxy-3-isopropyl-9-(methoxymethyl)-6,10a-dimethyl-1,2,4,5,6,6a,7,8,9,10a-decahydrodicyclopenta[a,d][8]annulen-4-yl alpha-D-glucopyranoside**	-0.089407	0.051523

**Gly-l-pro**	-0.096077	-0.025308
**4-Morpholinylacetic acid**	-0.060317	0.03708
**Adenine**	-0.066915	-0.09818
**Cucurbitacin A**	-0.061767	-0.099883
**BILA 2185BS**	-0.091391	0.02431
**Leukotriene D4**	-0.056505	-0.099353
**MCPB**	-0.01827	0.13317
**(Z)-Norendoxifen.1**	-0.025976	0.097109
**5,6-Dihydrothymidine**	-0.093553	0.040096
**Hypoxanthin**	0.029034	0.10794
**8-METHOXYKYNURENIC ACID**	0.022088	0.13154
**Telmisartan**	-0.086028	0.059469
**Linifanib**	-0.08257	0.066345
**ZV4**	-0.059118	0.019125
**Methohexital**	-0.090414	-0.052875
**MFCD00888473**	0.070544	-0.038482
**Ladostigil**	-0.036511	0.12429
**Valyl-4-hydroxyproline**	-0.049745	0.04763
**C8-Carnitine**	-0.03994	0.12323
**carnosine**	-0.039619	0.12034
**Aprobarbital**	0.077284	-0.062897
**Desmeninol**	0.081515	0.038173
**2-Phenylethyl D-glucopyranoside**	0.082432	0.068842
**FG7175000**	0.067116	-0.084252
**Kynurenic acid**	0.097636	-0.012947
**Acetyl-L-methionine**	0.061852	0.10551
**Hypoxanthin.1**	0.074586	0.08712
**(5alpha,7E)-7-Benzylidene-17-(cyclopropylmethyl)-3,14-dihydroxy-4,5-epoxymorphinan-6-one**	-0.043425	0.12045
**4-(1,2-Dihydroxy-2-propanyl)-1-methyl-1,2-cyclohexanediol**	0.082136	0.050279
**3-Oxotetradecanoic acid**	0.038298	0.092813
**Dodecylamine**	0.030519	0.089505
**1,3,7-Octanetriol**	0.050519	0.086457
**Androsterone glucuronide**	0.042353	0.12023
**2704846**	0.087087	0.04765

**(2S,4aS,6R,8aS)-6-[2-(beta-D-Glucopyranosyloxy)-2-propanyl]-8a-methyl-4-methylenedecahydro-2-naphthalenyl 6-O-[(2R,3R,4R)-3,4-dihydroxy-4-(hydroxymethyl)tetrahydro-2-furanyl]-beta-D-glucopyranoside**	0.010963	0.1336
**(10E,12Z)-9-Hydroperoxy-10,12-octadecadienoic acid**	0.081215	0.059637
**Biacetyl**	0.087466	0.040094
**(4S)-4-[(6-Carboxyhexanoyl)oxy]-4-(trimethylammonio)butanoate**	0.086745	0.060483
**9-Decenoylcarnitine**	-0.032479	0.12715
**MFCD00059633**	-0.014351	0.10686
**Indole**	-0.065927	0.099427
**12-Hydroxylauric acid**	0.079781	0.069666
**Arabinosylhypoxanthine**	0.014534	0.13305
**(1Z,3R,5E,8S,9S,10R)-N-[(Z)-2-(3-Chloro-4-hydroxyphenyl)vinyl]-3,9-dihydroxy-2,4-dimethoxy-6,8,10-trimethyl-7-oxo-5-tetradecenimidic acid**	0.096737	-0.024396
**putrescine**	-0.0010389	0.11567
**2,4-dimethyl-4,5-dihydro-1h-imidazole**	-0.067822	-0.094381
**gamma-Aminobutyric acid**	0.097483	-0.010552
**Choline.1**	0.068688	-0.045947
**MA4000000**	-0.068381	-0.095032
**Oryzalin metabolite**	-0.092319	-0.043685
**agmatine**	-0.092261	-0.02539
**Aminolevulinic acid**	-0.0676	-0.094852
**4-Morpholinylacetic acid.1**	-0.092355	-0.010233
**DL-Lysine**	0.0068647	-0.014035
**DL-Mevalonic acid**	-0.013122	0.026029
**Guanine.1**	-0.091823	-0.043575
**(2S,3S)-2,3-dihydro-3-hydroxyanthranilic acid zwitterion**	-0.066027	0.00084766
**2,2,6,6-Tetramethyl-1-piperidinol (TEMPO).1**	-0.058559	0.032801
**4-Methylcarbostyril.1**	-0.0045117	-0.1289
**n-phenethyl acetamide**	-0.049524	0.10144
**Uric Acid**	-0.012299	0.11873
**N(1)-acetylspermidine**	-0.043459	0.11989
**meglumine**	0.075372	-0.043552
**Alanylclavam**	-0.083324	0.0018514
**3,6,8-Trimethylallantoin**	-0.06311	0.099881
**5-(N,N-Dimethylcarbamimidamido)-2-oxopentanoic acid**	-0.088139	-0.035223

**5-Methoxy-3-indoleaceate**	-0.035446	0.12388
**N(4)-phosphoagmatine**	0.033056	0.073639
**2-{[(2S)-1-Methoxy-1-oxo-2-hexanyl]amino}-2-oxoethanediazonium**	-0.06656	-0.0023274
**2-C-methylerythritol 4-phosphate**	0.037033	0.064625
**2046365**	-0.065889	0.085653
**carnosine.1**	-0.083587	0.057853
**nylon cyclic dimer.1**	0.094477	0.023098
**Traumatic Acid.1**	-0.065747	-0.019014
**fructosylglycine**	0.034629	-0.070776
**1-Pentofuranosyl-2,4(1H,3H)-pyrimidinedione**	-0.012624	-0.058038
**Isopimpinellin**	-0.018102	0.068336
**Pindolol**	0.093918	0.034333
**Djenkolic Acid**	0.004306	0.1319
**2949.1**	-0.066165	0.025996
**L-Hexanoylcarnitine.1**	-0.077502	0.017143
**Ulodesine**	0.085044	0.047397
**S-(5-deoxy-beta-D-ribos-5-yl)-L-homocysteine**	0.044784	0.12088
**2-(2-Carboxyethyl)-4-methyl-5-pentyl-3-furoic acid**	-0.06714	-0.0015602
**4-[(3S,4R)-4-(4-Fluorophenyl)-3-hexanyl]phenol**	-0.058904	0.040217
**Gln-Gln**	0.031811	0.12041
**gamma-Glu-gln**	-0.091221	0.047156
**Abacavir**	-0.089339	0.059393
**imazamethabenz-methyl**	-0.071868	0.0057534
**8722455**	-0.041282	0.037001
**Embelin.1**	-0.014775	0.0068571
**L-Cysteinylglycine disulfide**	-0.068692	0.097009
**Tetraneurin A.1**	-0.049596	-0.087539
**Valclavam**	-0.033649	0.12376
**terameprocol**	-0.097456	0.0035623
**Bisacodyl.1**	-0.093705	0.043924
**Colcemid**	-0.082227	-0.049308
**Linifanib.1**	-0.092432	0.043523
**Dasolampanel**	-0.099303	-0.0065336
**tetrofosmin**	-0.061335	0.092056

**Bortezomib**	-0.030457	0.096175
**myxochelin B**	-0.088871	-0.054307
**gamma-Glutamyl-gamma-glutamylglutamic acid**	0.020353	0.039352
**1-palmitoylglycerone 3-phosphate**	0.077109	0.07181
**Aderbasib**	-0.082735	0.070174
**Leukotriene E3.1**	-0.087882	0.056665
**Hydrocortisone Valerate.1**	0.051181	0.063122
**Suvorexant**	-0.088465	-0.056694
**diphenoxylate**	0.075963	0.065934
**?-FNA**	-0.036774	-0.084865
**Sibiromycin**	-0.097912	-0.020381
**Telmisartan.1**	-0.092622	0.0041666
**7-[1-Formyl-6-hydroxy-6-(hydroxymethyl)bicyclo[3.2.1]oct-2-yl]-3a,7-dimethyl-3-oxooctahydro-2-benzofuran-1-yl hexopyranoside**	0.093712	-0.037733
**Zuclopenthixol decanoate.1**	-0.097211	0.015898
**Desoxymycin**	-0.096946	0.0094512
**BILA 2185BS.1**	-0.098877	-0.0021012
**D-gamma-Glutamyl-S-[(5Z,8beta,12E,15S)-1,15-dihydroxy-1,11-dioxoprosta-5,12-dien-9-yl]-L-cysteinylglycine.1**	-0.094753	0.040921
**Callichiline.1**	-0.098817	-0.0015085
**(2'S)-Deoxymyxol 2'-alpha-L-fucoside.1**	0.0091112	0.091487
