# Supplementary material for: Potential Probiotic Bacillus subtilis Isolated from a Novel Niche Exhibits Broad Range Antibacterial Activity and Causes Virulence and Metabolic Dysregulation in Enterotoxic E. coli
Source: Microorganisms. 2021 Jul 12;9(7):1483. doi: 10.3390/microorganisms9071483 (PMC8307078; doi:10.3390/microorganisms9071483)
Supplement: Supplementary file 1 [file microorganisms-09-01483-s001.zip › Table S3.pdf]

Table S3. Significant metabolites emerging in Co-culture and mono-cultures of CP9 and ETEC assessed by One-way ANOVA & post-hoc Tests.

Metabolite	p.value	FDR	Fisher's LSD
**Adenine**	5.6602e-11	1.1264e-08	CP9 - Co-culture; Co-culture - ETEC; CP9 - ETEC
**Levulinic acid**	2.5564e-10	2.5436e-08	CP9 - Co-culture; ETEC - Co-culture; CP9 - ETEC
**C8-Carnitine**	1.2239e-09	8.1187e-08	Co-culture - CP9; Co-culture - ETEC; CP9 - ETEC
**L-Cysteinylglycine disulfide**	2.1492e-09	1.0692e-07	Co-culture - CP9; Co-culture - ETEC; CP9 - ETEC
**Guanine**	6.1938e-09	2.4651e-07	CP9 - Co-culture; Co-culture - ETEC; CP9 - ETEC
**(5alpha,7E)-7-Benzylidene-17-(cyclopropylmethyl)-3,14-dihydroxy-4,5-epoxymorphinan-6-one**	8.9862e-09	2.9804e-07	Co-culture - CP9; Co-culture - ETEC; CP9 - ETEC
**N(1)-acetylspermidine**	1.8196e-08	5.1728e-07	Co-culture - CP9; Co-culture - ETEC; CP9 - ETEC
**Betamethasone dipropionate**	2.7445e-08	6.8269e-07	CP9 - Co-culture; ETEC - Co-culture; ETEC - CP9
**Bisacodyl.1**	4.3196e-08	8.425e-07	Co-culture - CP9; Co-culture - ETEC; CP9 - ETEC
**3-hydroxydodecanoylcarnitine**	4.502e-08	8.425e-07	CP9 - Co-culture; Co-culture - ETEC; CP9 - ETEC
**5,6-Dihydrothymidine**	4.6571e-08	8.425e-07	Co-culture - CP9; Co-culture - ETEC; CP9 - ETEC
**9-Decenoylcarnitine**	5.6871e-08	9.4311e-07	Co-culture - CP9; Co-culture - ETEC
**Zuclopenthixol decanoate**	6.6645e-08	1.0101e-06	Co-culture - CP9; Co-culture - ETEC; CP9 - ETEC
**Leukotriene C4**	7.2371e-08	1.0101e-06	Co-culture - CP9; Co-culture - ETEC; CP9 - ETEC
**Kynurenic acid**	7.6138e-08	1.0101e-06	Co-culture - CP9; ETEC - Co-culture; ETEC - CP9
**gamma-Aminobutyric acid**	9.8083e-08	1.2199e-06	Co-culture - CP9; ETEC - Co-culture; ETEC - CP9
**Arabinosylhypoxanthine**	1.0667e-07	1.2487e-06	Co-culture - CP9; Co-culture - ETEC; ETEC - CP9
**Zuclopenthixol decanoate.1**	1.1341e-07	1.2538e-06	Co-culture - ETEC; CP9 - ETEC
**D-gamma-Glutamyl-S-[(5Z,8beta,12E,15S)-1,15-dihydroxy-1,11-dioxoprosta-5,12-dien-9-yl]-L-cysteinylglycine**	1.6916e-07	1.7718e-06	Co-culture - CP9; Co-culture - ETEC; CP9 - ETEC
**Callichiline.1**	1.8365e-07	1.8273e-06	CP9 - Co-culture; Co-culture - ETEC; CP9 - ETEC
**myxochelin B**	2.4722e-07	2.3427e-06	CP9 - Co-culture; Co-culture - ETEC; CP9 - ETEC
**Sibiromycin**	2.7447e-07	2.4827e-06	CP9 - Co-culture; Co-culture - ETEC; CP9 - ETEC
**Guanine.1**	3.1204e-07	2.6999e-06	CP9 - Co-culture; Co-culture - ETEC; CP9 - ETEC
**Naloxegol**	3.5082e-07	2.8061e-06	Co-culture - CP9; Co-culture - ETEC; CP9 - ETEC

**N-(tert-Butoxycarbonyl)-L-leucine**	3.6605e-07	2.8061e-06	CP9 - Co-culture; Co-culture - ETEC; CP9 - ETEC
**gamma-Glu-gln**	3.6663e-07	2.8061e-06	Co-culture - CP9; Co-culture - ETEC; CP9 - ETEC
**(1Z,3R,5E,8S,9S,10R)-N-[(Z)-2-(3-Chloro-4-hydroxyphenyl)vinyl]-3,9-dihydroxy-2,4-dimethoxy-6,8,10-trimethyl-7-oxo-5-tetradecenimidic acid**	4.2936e-07	3.1645e-06	ETEC - Co-culture; ETEC - CP9
**D-gamma-Glutamyl-S-[(5Z,8beta,12E,15S)-1,15-dihydroxy-1,11-dioxoprosta-5,12-dien-9-yl]-L-cysteinylglycine.1**	4.8568e-07	3.4518e-06	Co-culture - CP9; Co-culture - ETEC; CP9 - ETEC
**(Hydroxyethyl)methacrylate**	5.185e-07	3.558e-06	CP9 - Co-culture; ETEC - Co-culture; CP9 - ETEC
**BILA 2185BS.1**	6.6157e-07	4.3205e-06	CP9 - Co-culture; Co-culture - ETEC; CP9 - ETEC
**Darifenacin**	6.7305e-07	4.3205e-06	CP9 - Co-culture; ETEC - Co-culture
**Abacavir**	7.3468e-07	4.5688e-06	Co-culture - CP9; Co-culture - ETEC; CP9 - ETEC
**(3S,6S,9S,14aR)-9-[(2S)-2-Butanyl]-6-[(1-methoxy-1H-indol-3-yl)methyl]-3-(6-oxooctyl)decahydropyrrolo[1,2-a][1,4,7,10]tetraazacyclododecine-1,4,7,10-tetrone**	8.3749e-07	5.0503e-06	Co-culture - CP9; Co-culture - ETEC; CP9 - ETEC
**Aderbasib**	1.1094e-06	6.4935e-06	Co-culture - CP9; Co-culture - ETEC; CP9 - ETEC
**L-Hexanoylcarnitine**	1.1895e-06	6.7632e-06	CP9 - Co-culture; Co-culture - ETEC; CP9 - ETEC
**carnosine**	1.4729e-06	7.963e-06	Co-culture - CP9; Co-culture - ETEC
**MCPB**	1.4806e-06	7.963e-06	Co-culture - CP9; Co-culture - ETEC; ETEC - CP9
**Indole**	1.6961e-06	8.8822e-06	Co-culture - CP9; Co-culture - ETEC
**5-Methoxy-3-indoleaceate**	2.0834e-06	1.0631e-05	Co-culture - CP9; Co-culture - ETEC
**Methohexital**	2.2147e-06	1.1018e-05	CP9 - Co-culture; Co-culture - ETEC; CP9 - ETEC
**spironolactone**	2.2715e-06	1.1025e-05	Co-culture - CP9; Co-culture - ETEC; CP9 - ETEC
**Ladostigil**	2.625e-06	1.2201e-05	Co-culture - CP9; Co-culture - ETEC
**Djenkolic Acid**	2.6363e-06	1.2201e-05	Co-culture - CP9; Co-culture - ETEC; ETEC - CP9
**Linifanib.1**	2.7968e-06	1.2649e-05	Co-culture - CP9; Co-culture - ETEC; CP9 - ETEC
**Mavik**	3.8788e-06	1.7153e-05	Co-culture - ETEC; CP9 - ETEC
**Astemizole**	3.9896e-06	1.7259e-05	CP9 - Co-culture; Co-culture - ETEC; CP9 - ETEC
**Dexamethasone beloxil**	4.1056e-06	1.7383e-05	Co-culture - ETEC; CP9 - ETEC
**Valclavam**	4.2569e-06	1.7648e-05	Co-culture - CP9; Co-culture - ETEC
**Ala-Tyr**	4.9104e-06	1.9942e-05	CP9 - Co-culture; Co-culture - ETEC; CP9 - ETEC
**3-[(2,6-Dimethylheptanoyl)oxy]-4-(trimethylammonio)butanoate**	5.3822e-06	2.1421e-05	Co-culture - CP9; Co-culture - ETEC; CP9 - ETEC
**2-methylbutyrylcarnitine**	6.0876e-06	2.3754e-05	CP9 - Co-culture; Co-culture - ETEC; CP9 - ETEC
**7-[1-Formyl-6-hydroxy-6-(hydroxymethyl)bicyclo[3.2.1]oct-2-yl]-3a,7-dimethyl-3-oxooctahydro-2-benzofuran-1-yl hexopyranoside**	6.6241e-06	2.535e-05	ETEC - Co-culture; ETEC - CP9

**(1S,4R,5R,6R,6aS,9S,9aE,10aR)-1,5-Dihydroxy-9-(hydroxymethyl)-3-isopropyl-6,10a-dimethyl-1,2,4,5,6,6a,7,8,9,10a-decahydrodicyclopenta[a,d][8]annulen-4-yl alpha-D-glucopyranoside**	6.7886e-06	2.5351e-05	CP9 - Co-culture; Co-culture - ETEC; CP9 - ETEC
**Leukotriene E3.1**	6.8791e-06	2.5351e-05	Co-culture - CP9; Co-culture - ETEC; CP9 - ETEC
**MA4000000**	7.5516e-06	2.7323e-05	CP9 - Co-culture; CP9 - ETEC
**Atosiban**	7.8695e-06	2.7965e-05	CP9 - Co-culture; Co-culture - ETEC; CP9 - ETEC
**8-METHOXYKYNURENIC ACID**	1.0513e-05	3.6704e-05	Co-culture - CP9; Co-culture - ETEC; ETEC - CP9
**[(2R,3R,4E,6E,9R,11S,12S,13S,14E)-2-Ethyl-9,11,13-trimethyl-8,16-dioxo-12-{[3,4,6-trideoxy-3-(dimethylamino)-beta-D-xylo-hexopyranosyl]oxy}oxacyclohexadeca-4,6,14-trien-3-yl]methyl 6-deoxy-beta-D-allo pyranoside**	1.1344e-05	3.8921e-05	CP9 - Co-culture; Co-culture - ETEC; CP9 - ETEC
**(2S,4aS,6R,8aS)-6-[2-(beta-D-Glucopyranosyloxy)-2-propanyl]-8a-methyl-4-methylenedecahydro-2-naphthalenyl 6-O-[(2R,3R,4R)-3,4-dihydroxy-4-(hydroxymethyl)tetrahydro-2-furanyl]-beta-D-glucopyranoside**	1.6167e-05	5.319e-05	Co-culture - CP9; Co-culture - ETEC; ETEC - CP9
**4-Methylcarbostyril**	1.6242e-05	5.319e-05	CP9 - Co-culture; ETEC - Co-culture; CP9 - ETEC
**Dasolampanel**	1.6304e-05	5.319e-05	CP9 - Co-culture; Co-culture - ETEC; CP9 - ETEC
**Telmisartan**	2.0387e-05	6.5434e-05	Co-culture - CP9; Co-culture - ETEC; CP9 - ETEC
**12-Hydroxylauric acid**	2.6076e-05	8.2367e-05	Co-culture - CP9; ETEC - Co-culture; ETEC - CP9
**GLIMEPIRIDE, CIS-**	2.6526e-05	8.2393e-05	CP9 - Co-culture; Co-culture - ETEC; CP9 - ETEC
**Aminolevulinic acid**	2.6912e-05	8.2393e-05	CP9 - Co-culture; CP9 - ETEC
**S-(5-deoxy-beta-D-ribos-5-yl)-L-homocysteine**	3.399e-05	0.00010248	Co-culture - CP9; Co-culture - ETEC; ETEC - CP9
**Linifanib**	3.4751e-05	0.00010321	Co-culture - CP9; Co-culture - ETEC; CP9 - ETEC
**Suvorexant**	3.5456e-05	0.00010376	CP9 - Co-culture; Co-culture - ETEC; CP9 - ETEC
**7-Sulfocholic acid**	3.6472e-05	0.00010519	CP9 - Co-culture; Co-culture - ETEC; CP9 - ETEC
**chivosazole A**	4.0183e-05	0.00011423	CP9 - Co-culture; Co-culture - ETEC; CP9 - ETEC
**(1S,4R,5R,6R,6aS,9S,9aE,10aR)-1,5-Dihydroxy-3-isopropyl-9-(methoxymethyl)-6,10a-dimethyl-1,2,4,5,6,6a,7,8,9,10a-decahydrodicyclopenta[a,d][8]annulen-4-yl alpha-D-glucopyranoside**	4.4742e-05	0.00012534	Co-culture - ETEC; CP9 - ETEC
**N-[(10Z)-3-sec-Butyl-7-isobutyl-5,8-dioxo-2-oxa-6,9-diazabicyclo[10.2.2]hexadeca-1(14),10,12,15-tetraen-4-yl]-1-methylprolinamide**	4.535e-05	0.00012534	Co-culture - ETEC; CP9 - ETEC
**Desoxymycin**	4.6087e-05	0.00012564	Co-culture - ETEC; CP9 - ETEC
**Cucurbitacin A**	4.96e-05	0.00013338	CP9 - Co-culture; CP9 - ETEC
**Acetyl-L-methionine**	5.8373e-05	0.00015488	Co-culture - CP9; ETEC - CP9

**3,6,8-Trimethylallantoin**	6.1123e-05	0.00016005	Co-culture - CP9; Co-culture - ETEC; CP9 - ETEC
**Arg-Trp**	6.2628e-05	0.00016186	CP9 - Co-culture; Co-culture - ETEC; CP9 - ETEC
**Gln-Gln**	0.00010342	0.00026385	Co-culture - CP9; Co-culture - ETEC; ETEC - CP9
**4-Methylcarbostyril.1**	0.000111	0.00027465	CP9 - Co-culture; ETEC - Co-culture; CP9 - ETEC
**Lithocholic acid taurine conjugate**	0.00011131	0.00027465	Co-culture - ETEC; CP9 - ETEC
**2-methylbutyrylcarnitine.1**	0.00011179	0.00027465	CP9 - Co-culture; Co-culture - ETEC; CP9 - ETEC
**Enviradene**	0.0001178	0.00028589	Co-culture - ETEC; CP9 - ETEC
**carnosine.1**	0.00012389	0.00029703	Co-culture - CP9; Co-culture - ETEC; CP9 - ETEC
**Melagatran**	0.00015017	0.00035575	Co-culture - CP9; Co-culture - ETEC; CP9 - ETEC
**perphenazine decanoate**	0.00016864	0.00039406	Co-culture - ETEC; CP9 - ETEC
**BILA 2185BS**	0.0001703	0.00039406	Co-culture - ETEC; CP9 - ETEC
**agmatine**	0.00021269	0.0004865	CP9 - Co-culture; Co-culture - ETEC; CP9 - ETEC
**Callichiline**	0.00021896	0.00049514	Co-culture - CP9; Co-culture - ETEC; CP9 - ETEC
**Telmisartan.1**	0.00024592	0.00054988	Co-culture - ETEC; CP9 - ETEC
**Gly-l-pro**	0.00027361	0.00060498	CP9 - Co-culture; Co-culture - ETEC; CP9 - ETEC
**2-Phenylethyl D-glucopyranoside**	0.00032936	0.00072024	Co-culture - CP9; ETEC - Co-culture; ETEC - CP9
**Androsterone glucuronide**	0.00036076	0.00078034	Co-culture - CP9; ETEC - CP9
**trilobolide**	0.00040307	0.00086248	CP9 - Co-culture; Co-culture - ETEC; CP9 - ETEC
**terameprocol**	0.00043129	0.00091304	Co-culture - ETEC; CP9 - ETEC
**2,4-dimethyl-4,5-dihydro-1h-imidazole**	0.00045216	0.00094715	CP9 - Co-culture; CP9 - ETEC
**Hypoxanthin.1**	0.00047003	0.00097434	Co-culture - CP9; ETEC - CP9
**(4S)-4-[(6-Carboxyhexanoyl)oxy]-4-(trimethylammonio)butanoate**	0.00051086	0.0010481	Co-culture - CP9; ETEC - Co-culture; ETEC - CP9
**1-O-[(3alpha,5beta,7alpha)-3,7-Dihydroxy-24-oxocholan-24-yl]-beta-D-galactopyranose**	0.00063098	0.0012813	Co-culture - ETEC; CP9 - ETEC
**Pindolol**	0.00085913	0.0017269	Co-culture - CP9; ETEC - Co-culture; ETEC - CP9
**Leu-Gly-Pro**	0.00087867	0.0017485	CP9 - Co-culture; Co-culture - ETEC; CP9 - ETEC
**Oryzalin metabolite**	0.00089263	0.0017486	CP9 - Co-culture; Co-culture - ETEC; CP9 - ETEC
**5-(N,N-Dimethylcarbamimidamido)-2-oxopentanoic acid**	0.00089624	0.0017486	CP9 - Co-culture; Co-culture - ETEC; CP9 - ETEC
**3-[(3-Hydroxyundecanoyl)oxy]-4-(trimethylammonio)butanoate**	0.00092045	0.0017783	Co-culture - CP9; Co-culture - ETEC
**(3beta,5beta)-24-Hydroxy-24-oxocholan-3-yl beta-D-glucopyranosiduronic acid**	0.0010426	0.001995	Co-culture - CP9; Co-culture - ETEC; CP9 - ETEC
**1-(4-Butylphenyl)-6,6-dimethyl-1,6-dihydro-1,3,5-triazine-2,4-diamine**	0.0010929	0.0020713	Co-culture - CP9; Co-culture - ETEC; CP9 - ETEC

**n-phenethyl acetamide**	0.0011712	0.0021988	Co-culture - CP9; Co-culture - ETEC
**Bisacodyl**	0.001279	0.0023787	Co-culture - ETEC; CP9 - ETEC
**nylon cyclic dimer.1**	0.0013413	0.0024714	ETEC - Co-culture; ETEC - CP9
**1-palmitoylglycerone 3-phosphate**	0.0016616	0.0030336	Co-culture - CP9; ETEC - CP9
**Methionylleucine**	0.0020577	0.0037226	Co-culture - ETEC; CP9 - ETEC
**2046365**	0.0021152	0.0037921	Co-culture - CP9; Co-culture - ETEC; CP9 - ETEC
**LT9970000**	0.0029438	0.0052304	Co-culture - CP9; Co-culture - ETEC
**Leukotriene D4**	0.0029846	0.0052561	CP9 - Co-culture; CP9 - ETEC
**tetrofosmin**	0.0030725	0.0053634	Co-culture - CP9; Co-culture - ETEC; CP9 - ETEC
**Desmeninol**	0.0031929	0.0055251	Co-culture - CP9; ETEC - Co-culture; ETEC - CP9
**Uric Acid**	0.0034036	0.0058263	Co-culture - CP9; Co-culture - ETEC
**FG7175000**	0.0034255	0.0058263	CP9 - Co-culture; ETEC - Co-culture; ETEC - CP9
**(Hydroxyethyl)methacrylate.1**	0.0034827	0.0058734	Co-culture - CP9; ETEC - Co-culture; ETEC - CP9
**(Z)-Norendoxifen**	0.0053935	0.0090165	Co-culture - ETEC; CP9 - ETEC
**Rizatriptan**	0.0054371	0.0090165	Co-culture - ETEC; CP9 - ETEC
**putrescine**	0.0071156	0.011703	Co-culture - CP9; Co-culture - ETEC
**2704846**	0.0086956	0.014184	Co-culture - CP9; ETEC - CP9
**Aprobarbital**	0.0099312	0.016068	ETEC - Co-culture; ETEC - CP9
**4-Morpholinylacetic acid.1**	0.010936	0.017551	Co-culture - ETEC; CP9 - ETEC
**nylon cyclic dimer**	0.011263	0.017931	ETEC - CP9
**(2R)-1-[(Hydroxy{[(1s,3R)-2,3,4,5,6-pentahydroxycyclohexyl]oxy}phosphoryl)oxy]-3-(palmitoyloxy)-2-propanyl (5Z,8Z,11Z)-5,8,11-icosatrienoate**	0.011981	0.018923	Co-culture - CP9; ETEC - CP9
**(2'S)-Deoxymyxol 2'-alpha-L-fucoside**	0.012294	0.019264	Co-culture - CP9; ETEC - CP9
**Biacetyl**	0.013225	0.020561	ETEC - CP9
**(10E,12Z)-9-Hydroperoxy-10,12-octadecadienoic acid**	0.01498	0.023108	Co-culture - CP9; ETEC - CP9
**Ulodesine**	0.015883	0.024313	ETEC - CP9
**Cifenline**	0.017734	0.026939	ETEC - CP9
**3-Oxotetradecanoic acid**	0.018555	0.027973	Co-culture - CP9; ETEC - CP9
**Colcemid**	0.019424	0.029063	CP9 - ETEC
**Choline**	0.020141	0.029911	Co-culture - CP9; ETEC - CP9
**diphenoxylate**	0.023558	0.034726	Co-culture - CP9; ETEC - CP9
**MFCD00059633**	0.023742	0.03474	Co-culture - CP9; Co-culture - ETEC
**4-(1,2-Dihydroxy-2-propanyl)-1-methyl-1,2-cyclohexanediol**	0.024987	0.036295	ETEC - CP9
**SECONAL**	0.02887	0.041632	Co-culture - CP9; ETEC - CP9
**Hypoxanthin**	0.032554	0.046606	Co-culture - CP9
**Prednisolone tebutate**	0.033762	0.047758	Co-culture - ETEC
**Leukotriene E3**	0.033839	0.047758	Co-culture - ETEC
