# Supplementary material for: Potential Probiotic Bacillus subtilis Isolated from a Novel Niche Exhibits Broad Range Antibacterial Activity and Causes Virulence and Metabolic Dysregulation in Enterotoxic E. coli
Source: Microorganisms. 2021 Jul 12;9(7):1483. doi: 10.3390/microorganisms9071483 (PMC8307078; doi:10.3390/microorganisms9071483)
Supplement: Supplementary file 1 [file microorganisms-09-01483-s001.zip › Table S4.pdf]

Table S4. Variable Importance in projection (VIP) scores across PLS-DA components indicating scale of variable metabolite concentration in mono-cultures and co-culture of CP9 and ETEC.

Metabolites	VIP Scores (>1)	
	**Comp. 1**	**Comp. 2**
**L-Cysteinylglycine disulfide**	1.5642	1.5136
**Indole**	1.5536	1.5057
**Aderbasib**	1.5448	1.4852
**3,6,8-Trimethylallantoin**	1.5298	1.4847
**Linifanib**	1.5179	1.4589
**Melagatran**	1.5172	1.4729
**Abacavir**	1.5147	1.454
**spironolactone**	1.5097	1.4494
**Telmisartan**	1.4998	1.4401
**Callichiline**	1.4993	1.44
**Leukotriene E3.1**	1.4956	1.4358
**carnosine.1**	1.4944	1.4362
**D-gamma-Glutamyl-S-[(5Z,8beta,12E,15S)-1,15-dihydroxy-1,11-dioxoprosta-5,12-dien-9-yl]-L-cysteinylglycine**	1.491	1.4312
**Zuclopenthixol decanoate**	1.4877	1.428
**(3S,6S,9S,14aR)-9-[(2S)-2-Butanyl]-6-[(1-methoxy-1H-indol-3-yl)methyl]-3-(6-oxooctyl)decahydropyrrolo[1,2-a][1,4,7,10]tetraazacyclododecine-1,4,7,10-tetrone**	1.4855	1.4261
**1-(4-Butylphenyl)-6,6-dimethyl-1,6-dihydro-1,3,5-triazine-2,4-diamine**	1.4731	1.4174
**3-[(2,6-Dimethylheptanoyl)oxy]-4-(trimethylammonio)butanoate**	1.4724	1.4402
**gamma-Glu-gln**	1.4715	1.4125
**2046365**	1.4688	1.4207
**Naloxegol**	1.4683	1.4096
**(3beta,5beta)-24-Hydroxy-24-oxocholan-3-yl beta-D-glucopyranosiduronic acid**	1.4674	1.4101
**Linifanib.1**	1.463	1.4045
**(1S,4R,5R,6R,6aS,9S,9aE,10aR)-1,5-Dihydroxy-3-isopropyl-9-(methoxymethyl)-6,10a-dimethyl-1,2,4,5,6,6a,7,8,9,10a-decahydrodicyclopenta[a,d][8]annulen-4-yl alpha-D-glucopyranoside**	1.459	1.4006
**tetrofosmin**	1.4509	1.4063
**FG7175000**	1.4488	1.399
**Methionylleucine**	1.4478	1.394
**LT9970000**	1.4475	1.4108
**(5alpha,7E)-7-Benzylidene-17-(cyclopropylmethyl)-3,14-dihydroxy-4,5-epoxymorphinan-6-one**	1.4459	1.4262
**D-gamma-Glutamyl-S-[(5Z,8beta,12E,15S)-1,15-dihydroxy-1,11-dioxoprosta-5,12-dien-9-yl]-L-cysteinylglycine.1**	1.4408	1.3844
**N(1)-acetylspermidine**	1.4443	1.4248

**Betamethasone dipropionate**	1.4408	1.4224
**5,6-Dihydrothymidine**	1.4407	1.3837
**Dexamethasone beloxil**	1.435	1.3777
**n-phenethyl acetamide**	1.4326	1.4083
**Leukotriene C4**	1.4209	1.3659
**7-[1-Formyl-6-hydroxy-6-(hydroxymethyl)bicyclo[3.2.1]oct-2-yl]-3a,7-dimethyl-3-oxooctahydro-2-benzofuran-1-yl hexopyranoside**	1.4202	1.3644
**C8-Carnitine**	1.4194	1.4042
**Bisacodyl**	1.4188	1.3625
**carnosine**	1.412	1.3994
**Rizatriptan**	1.3971	1.3433
**Ladostigil**	1.3933	1.3839
**Aprobarbital**	1.3853	1.3304
**1-O-[(3alpha,5beta,7alpha)-3,7-Dihydroxy-24-oxocholan-24-yl]-beta-D-galactopyranose**	1.3708	1.3164
**5-Methoxy-3-indoleaceate**	1.3661	1.3571
**9-Decenoylcarnitine**	1.3611	1.3578
**Valclavam**	1.3586	1.3543
**(1Z,3R,5E,8S,9S,10R)-N-[(Z)-2-(3-Chloro-4-hydroxyphenyl)vinyl]-3,9-dihydroxy-2,4-dimethoxy-6,8,10-trimethyl-7-oxo-5-tetradecenimidic acid**	1.3569	1.3068
**3-[(3-Hydroxyundecanoyl)oxy]-4-(trimethylammonio)butanoate**	1.3429	1.3343
**8-Methoxykynurenic acid**	1.3368	1.3216
**BILA 2185BS**	1.3299	1.2781
**Darifenacin**	1.284	1.2952
**Kynurenic acid**	1.2825	1.2397
**Gln-Gln**	1.2812	1.2803
**Prednisolone tebutate**	1.2685	1.2218
**gamma-Aminobutyric acid**	1.2656	1.2242
**Leukotriene E3**	1.262	1.247
**Mavik**	1.2505	1.211
**Choline.1**	1.2336	1.1859
**Enviradene**	1.2324	1.1926
**perphenazine decanoate**	1.2268	1.1872
**MCPB**	1.2177	1.2362
**Desoxymycin**	1.2173	1.183
**N-[(10Z)-3-sec-Butyl-7-isobutyl-5,8-dioxo-2-oxa-6,9-diazabicyclo[10.2.2]hexadeca-1(14),10,12,15-tetraen-4-yl]-1-methylprolinamide**	1.215	1.1748
**(Z)-Norendoxifen**	1.2115	1.1678
**meglumine**	1.2076	1.1597
**Astemizole**	1.2056	1.17
**terameprocol**	1.2018	1.1675
**Arabinosylhypoxanthine**	1.1994	1.1915

**Ala-Tyr**	1.1993	1.1635
**Lithocholic acid taurine conjugate**	1.1889	1.1512
**BILA 2185BS.1**	1.1778	1.1487
**Telmisartan.1**	1.1713	1.1349
**2-Amino-9,10-epoxy-8-oxodecanoic acid**	1.1503	1.1262
**MFCD00888473**	1.1471	1.1011
**Pulcherriminic acid**	1.1402	1.0945
**Dasolampanel**	1.1367	1.1149
**(Z)-Norendoxifen.1**	1.0869	1.0859
**Bortezomib**	1.0776	1.0615
**Uric Acid**	1.039	1.0652
**MFCD00059633**	1.0336	1.0203
**Sibiromycin**	1.0275	1.0208
**putrescine**	1.0175	1.0174
